# Functional Reorganization of the Default Mode Network across Chronic Pain Conditions

**DOI:** 10.1371/journal.pone.0106133

**Published:** 2014-09-02

**Authors:** Marwan N. Baliki, Ali R. Mansour, Alex T. Baria, A. Vania Apkarian

**Affiliations:** 1 Department of Physiology, Feinberg School of Medicine, Northwestern University, Chicago, Illinois, United States of America; 2 Department of Anesthesia, Feinberg School of Medicine, Northwestern University, Chicago, Illinois, United States of America; 3 Rehabilitation Institute of Chicago, Chicago, Illinois, United States of America; Hangzhou Normal University, China

## Abstract

Chronic pain is associated with neuronal plasticity. Here we use resting-state functional magnetic resonance imaging to investigate functional changes in patients suffering from chronic back pain (CBP), complex regional pain syndrome (CRPS) and knee osteoarthritis (OA). We isolated five meaningful resting-state networks across the groups, of which only the default mode network (DMN) exhibited deviations from healthy controls. All patient groups showed decreased connectivity of medial prefrontal cortex (MPFC) to the posterior constituents of the DMN, and increased connectivity to the insular cortex in proportion to the intensity of pain. Multiple DMN regions, especially the MPFC, exhibited increased high frequency oscillations, conjoined with decreased phase locking with parietal regions involved in processing attention. Both phase and frequency changes correlated to pain duration in OA and CBP patients. Thus chronic pain seems to reorganize the dynamics of the DMN and as such reflect the maladaptive physiology of different types of chronic pain.

## Introduction

Recent anatomical and functional imaging studies in humans are beginning to provide new insights into the brain reorganization associated with chronic pain. The notion that brain activity associated with chronic pain is localized to a definitive neural substrate that passively reflects peripheral and spinal changes following injury is no longer tenable.Instead, recent brain functional magnetic resonance imaging (fMRI) and MRI studies show that multiple chronic pain conditions are associated with metabolic changes within large-scale distributed networks involved in a plethora of sensory, motor, autonomic,cognitive, and emotional functions [1], [2].

It is critical to take into account brain activity that occurs in the absence of overt stimulation in order to better understand how the brain functions, reorganizes and adapts in the presence of chronic pain. The resting state techniques maps temporally synchronous, spatially distributed, spontaneous blood-oxygen level-dependent (BOLD) signal fluctuations at rest or, more accurately, in task-free settings [3] [4]. Since the brain of chronic pain patients is continuously processing spontaneous background pain and since presence of spontaneous pain interferes with other conscious or sub-conscious processes, such methods provide a powerful tool with the potential to detect fundamental aspects of brain pathophysiology associated with chronic clinical pain conditions.

To date changes in resting state networks (RSN) have been observed for different chronic pain conditions including chronic back pain (CBP), fibromyalgia, temporomandibular disorder and diabetic neuropathy [5], [6], [7], [8], [9], [10]. Initial observations indicate the default mode network (DMN) to be the primary network affected by chronic pain (see [7]). The DMN is constituted from a set of synchronous brain regions that are active at rest and deactivated during task performance [11]. While the exact functions of the DMN are not completely identified, elements of this network have been shown to participate in episodic memory [12], [13] and in monitoring the internal, in contrast to the external, environment for the detection of salient events, thus maintaining a background level of attention [11]. Furthermore, recent studies have shown than the DMN can modulate pain perception of acute nocious stimulti to oneslef or another through autonomic and antinociceptive descending modulation networks [14], [15].While the DMN has been shown to be disrupted in multiple chronic pain conditions, it remains unclear whether these changes mirror specific biological and physiological processes associated with different chronic pain phenotypes, or reflect a more ubiquitous reorganization of the brain resting state networks shared across chronic pain conditions. It is also unknown how these changes are related to, and interact with the pain specific regions that directly receive afferent input from peripheral nociceptive pathways, and which have been regularly shown to be involved in the processing and modulation of painful stimuli [16], [17].

Here we attempt to identify functional changes across different chronic pain conditions, elucidate the mechanisms underlying these changes, and relate them to disease etiology. To this aim, we investigate the functional connectivity of five well-established RSNs in patients suffering from CBP (n = 18), complex regional pain syndrome (CRPS, n = 19) and osteoarthritis (OA, n = 14) compared to healthy controls (n = 36). We also examine frequency content and phase relationships of BOLD oscillations of RSNs, which delineate the interrelationship between temporal and spatial changes in connectivity dynamics. Since chronic pain has been shown to be associated with specific decreased gray matter density [18], [19], we utilize a full factorial design to investigate differences in function after correcting for gray matter density changes. Thus we utilize multiple approaches in order to delineate the mechanisms underlying brain pathophysiology of chronic pain and to underscore the potential of resting state fMRI to provide clinically useful information.

## Material and Methods

### Subjects

Subjects that participated in this study were a subset from a previous study [18] and included 36 healthy subjects (24 females, 12 males; age: mean  = 41.36, range  = 21–70, S.E.M.  = 2.05 years), 18 CBP patients (5 females, 13 males; average age: mean  = 51.55, range = 32–62, S.E.M.  = 1.87 years), 19 CRPS patients (16 females, 3 males; average age: mean  = 40.94, range = 25–61, S.E.M.  = 2.45 years) and 14 OA patients (6 females, 8 males; average age: mean  = 58.29, range = 42–77, S.E.M.  = 2.64 years) All participants were right-handed, and gave informed consent to procedures approved by Northwestern University institutional review board (IRB) for protection of human subjects. Patients were diagnosed by a clinician and fulfilled the International Association for the Study of Pain (IASP) criteria, and had to satisfy a specific list of inclusion/exclusion criteria. Patients were excluded if they reported more than mild to moderate depression as defined by Beck's Depression Inventory (BDI) (BDI>19), or other chronic painful and psychiatric conditions, systemic disease, or history of head injury or coma. The demographic data and pain-related parameters for CBP, CRPS and OA patients are are presented in Table S1.

### Clinical parameters and medication

All patients completed the short-form of the McGill pain questionnaire (SF-MPQ) [20]. The main component of the SF-MPQ consists of 12 sensory and 4 affective descriptors; it also includes a visual analog scale (VAS) (0 =  no pain, 10 =  maximum imaginable pain) and pain duration. Depression scores for all subjects that participated in the study were assessed using BDI-ii. All questioners were given 1 hour prior to brain scanning. Drug consumption was quantified using the Medication Quantification Scale (MQS) [21], which reduces drugs used for different durations and doses to a single scalar. The MQS was used as a covariate of no interest to control for drug usage. However, inclusion of this covariate did not result in important changes in outcomes. A list of medications and corresponding MQS scores for each patient are presented in **Table S1**.

### Functional and structural imaging

All imaging data was acquired using a 3T Siemens Trio whole-body scanner with echo-planar imaging (EPI) capability using the standard 8-channel radio-frequency head coil. The anatomical and fMRI scans were collected during a single brain imaging session.

Functional scans (300 volumes, 12 mins) were acquired for all subjects. All participants had no task, but were instructed to stay alert and keep their eyes open for the duration of the scan. Images were obtained with the following parameters: Multi-slice T2*-weighted echo-planar images were repetition time TR  = 2.5 s, echo time TE  = 30 ms, flip angle  = 90°, FOV  = 256 mm, slice thickness  = 3 mm, in-plane resolution  = 64×64. The 40 slices covered the whole brain from the cerebellum to the vertex.

In addition to the functional scans, a T1-weighted anatomical MRI image was also acquired for each subject using the following parameters: TR  = 2.1 s, TE  = 4.38 ms, flip angle  = 8°, FOV  = 220 mm, slice thickness  = 1 mm, in-plane resolution  = 0.861×0.861 mm^2^ and number of sagittal slices  = 160.

### Voxel based morphometry (VBM)

Regional gray matter density was assessed with VBM using the optimized method and nonparametric statistical contrasts [22], [23] using FSL 4.0 software. The protocol included the following steps: first, a left-right symmetric study-specific gray matter template was built from 56 gray-matter-segmented native images (14 images were randomly selected from each group to minimize size of population bias). Custom images for each subject were generated by applying affine and deformation parameters obtained from normalizing the grey matter images, segmented in native space, to the custom template. Modulation was performed by multiplying voxel values by the Jacobian determinants derived from the spatial normalization step. Finally, images were smoothed with isotropic Gaussian kernel (sigma  = 3.5, FWHM  = 8 mm).

### fMRI preprocessing and ICN derivation

The pre-processing of each subject's time series of fMRI volumes was performed using the FMRIB Expert Analysis Tool (FEAT, [24], www.fmrib.ox.ac.uk/fsl) and encompassed: Discarding the first five volumes to allow for magnetic field stabilization; skull extraction using BET; slice time correction; motion correction; spatial smoothing using a Gaussian kernel of FWHM 5 mm; and high-pass temporal filtering (150 seconds). Several sources of noise, which may contribute to non-neuronal fluctuations, were removed from the data through linear regression. These included the six parameters obtained by rigid body correction of head motion, the global BOLD signal averaged over all voxels of the brain, signal from a ventricular region of interest, and signal from a region centered in the white matter.

Independent component network (ICN) analysis and statistical comparisons were performed following procedures outlined by Zhou and colleagues [25]. After preprocessing, functional images were concatenated into 4D files and entered into FSL 4.0 Melodic ICA software [26] to identify large-scale patterns of functional connectivity. Next, we used an automated template matching procedure to obtain subject-specific best-fit ICN maps. Goodness-of-fit was calculated by comparing each component from each subject to binarized group ICA maps built from 17 healthy young subjects (9 females, 8 males, mean age  = 26.5, range  = 19–40, S.E.M.  = 2.13 years, all right-handed) from a separate dataset that has been published previously [27]. These ICN templates (**Figure S1**) were thresholded at a *z*-score ≥4.0 to be comparable to the consistent ICNs published by Damoiseaux *et al.* (2006). Goodness-of-fit scores for each ICN was calculated by multiplying 1) the average *z*-score difference between voxels falling within the template and voxels falling outside the template; and 2) the difference in the percentage of positive *z*-score voxels inside and outside the template. [25]. Thus within the selected ICA component, each voxel's *z*-score represents the degree to which that voxel's time series correlates with the overall component time series.

Group differences in ICN connectivity was performed using random effects analyses on each subject's best-fit component images and a ‘full factorial’ F-test design implemented in FSL after correcting for gender and age effects (**Figure S1**). Statistical maps were corrected for multiple comparison using family wise error (FWE) cluster correction (p<0.01). In addition we re-analyzed each contrast after adding voxel-wise grey matter probability maps as covariates of no interest. Grey matter probability maps derived from voxel-based morphometry were registered to the same standard image space as the functional images and were re-sampled to equalize voxel sizes and image dimensions across the functional and structural data. Thus significant group differences at each voxel reflect focal connectivity reduction or enhancement relative to the associated overall ICN.

### Spectral power analysis

Spectral analysis was carried out using custom Matlab (The MathWorks, 2009) routines, and closely follows procedures outlined by Baria et al. [27]. Frequency power of the BOLD signal was determined using Welch's method and normalized by dividing by total power. The average power of each frequency band (low frequency: 0.01–0.05 Hz, mid frequency: 0.05–0.12 Hz and high frequency: 0.12–0.20 Hz) was calculated for each time series and subject. Group differences in spectral power for each frequency band was computed using an ANCOVA analysis with age and gender as confounding factors. Post hoc comparisons between each patient group and healthy controls were performed using a two-tailed Dunnett test.

### Phase analysis

Phase differences were carried out using custom Matlab (The MathWorks, 2009) routines. Voxel-wise phase differences were determined by first computing the instantaneous phase for a given voxel using Hilbert function and subtracting it from that of the DMN, which resulted in the instantaneous phase difference for every voxel in the brain to the DMN for a given subject. Individual subject phase difference maps were transformed into standard space using FLIRT [28], and multiplied by a standard gray matter mask and entered into a F-test design utilizing circular Watson-Williams parametric test. Phase differences between networks was obtained simply by subtracting time courses from one another and calculating the absolute value of the mean phase difference across time. Whole brain voxel-wise differences in the phase relationship to the DMN across groups was determined using an ANOVA (mixed effects analysis, f-zscore >3.0, corrected for multiple comparisons by FEW cluster threshold p<0.01).

### Region of interest (ROI) and BOLD analysis

The ROIs were fixed size, 10-mm-diameter spheres, centered at peak coordinates defined from ICA analysis and included the MPFC (x = −4, y = 58, z = 2), PreCu (x = 2, y = −56, z = 26), ACC (x = 2, y = 36, z = 22), right LP (x = 46,y = −60, z = 32), left IFG (x = −38,y = 10,z = −12) and left SMG (x = −56, y = −36, z = 26). An additional ROI in the INS (x = 42, y = 14, z = −6) was defined from the MPFC connectivity contrast map. In order to extract the BOLD signal for each ROI and subject, the ROIs were reverse-normalized and projected back into individual brain space and BOLD signal for the total trial duration was obtained by averaging the raw data for all voxels across a given ROI. BOLD time course was measured by calculating percent BOLD change (deviation from the mean for voxels within the ROI).

### Functional connectivity analysis

Brain MPFC correlation maps were identified using a well-validated method, see [29], [30]. Correlation maps were produced for the resting-state functional scans by first extracting the BOLD time course from the MPFC ROI and then computing correlation coefficient between its time course and the time variability of all other brain voxels. Correlation coefficients were converted to a normal distribution using the Fischer's z-transform. These values were then converted to z-scores (i.e. normalized correlation values) by dividing by the square root of the variance, estimated as 1/√(df-3), where df represents the degrees of freedom in our measurement (300 data points). Because the BOLD time course of consecutive samples are not statistically independent, the degrees of freedom were corrected by a factor of 2.86, in accordance to Bartlett theory (Jenkins and Watts, 1968), resulting in 300/2.86 = 104 degrees of freedom. A F-test was used to compute significant differences in correlations (Fischer's z-transformed values) across groups using a random effects analysis (z-score >3.0, cluster threshold P<0.01, FWE cluster-based corrected for multiple comparisons).

## Results

### The DMN exhibits altered spatial extent and connectivity properties in patients

In order to identify the RSNs of interest, we first performed a group independent component analysis (ICA) on an independent data set of healthy controls from a previously published study [27]. We selected five components that coincided with resting state networks most commonly observed, studied and described in previous reports [31], [32]. **Figure S1** shows these components, commonly denoted as the default mode, salience, sensorimotor, right frontoparietal and visual networks. Next, we used an automated template matching procedure to obtain subject-specific best-fit independent component map for each of the five networks for all patients and healthy controls as described by Zhou and colleagues [25].

**Figure 1:**
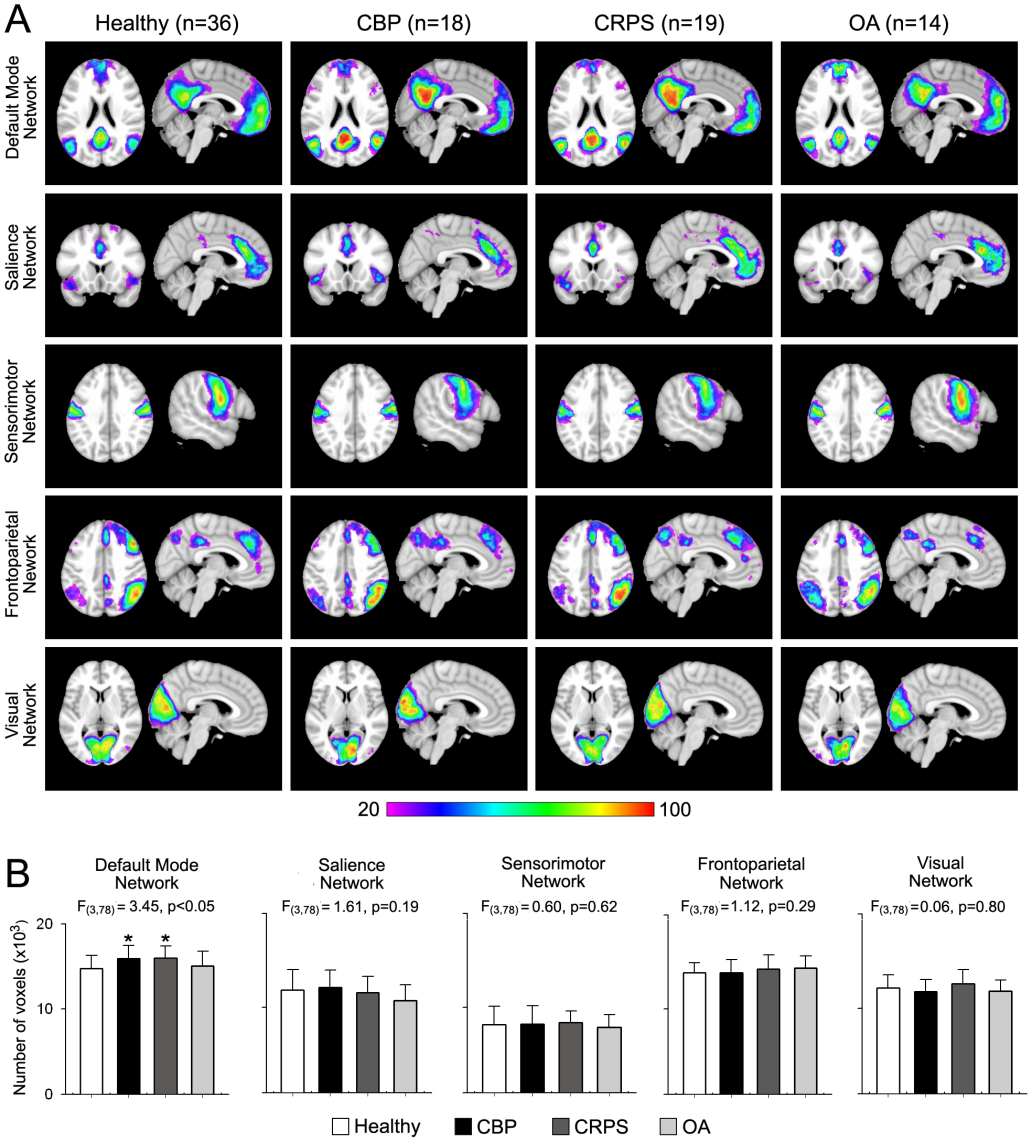
Spatial properties of resting state networks in three chronic pain patient groups and in healthy controls. (**A**) Percent spatial overlap of five resting state networks (RSNs) for healthy and pain patient groups. Colors represent percentage of subjects whose best fit component overlap at each voxel. Red denotes much overlap, while purple denotes little overlap. Overall, All RSNs show similar spatial representation across all groups with the exception of the default mode network (DMN), which exhibits larger overlap in the precuneus and posterior cingulate and less overlap in medial prefrontal cortex for CBP and CRPS groups. (**B**) Mean ± S.E.M. of number of voxels (z-score >3.0) of each RSN. The DMN is the only RSN that differs in size across groups (F_3,78_ = 3.45, p<0.05), with the CBP and CRPS groups having a larger DMN compared to controls (Post hoc test, *p<0.05 vs controls).

Spatial properties of the five RSNs for all groups are shown in **Figure 1A**. Group conjunction maps reveal analogous spatial representation across all groups (i.e. each of five the RSNs examined were identified in patients and healthy subjects). We first examined differences in size for all RSNs using an analysis of covariance (ANCOVA), with age and gender modeled as variables of no interest. **Figure 1B** show the mean ± S.E.M. of number of voxels within each RSN for all groups after registering the subject's best-fit component image to standard template (MNI space). Only the DMN showed significant differences across groups (F_3,78_ = 3.45, p<0.05) with the CBP and CRPS patients having a larger DMN compared to healthy controls (post-hoc p<0.05 for both groups).

**Figure 2:**
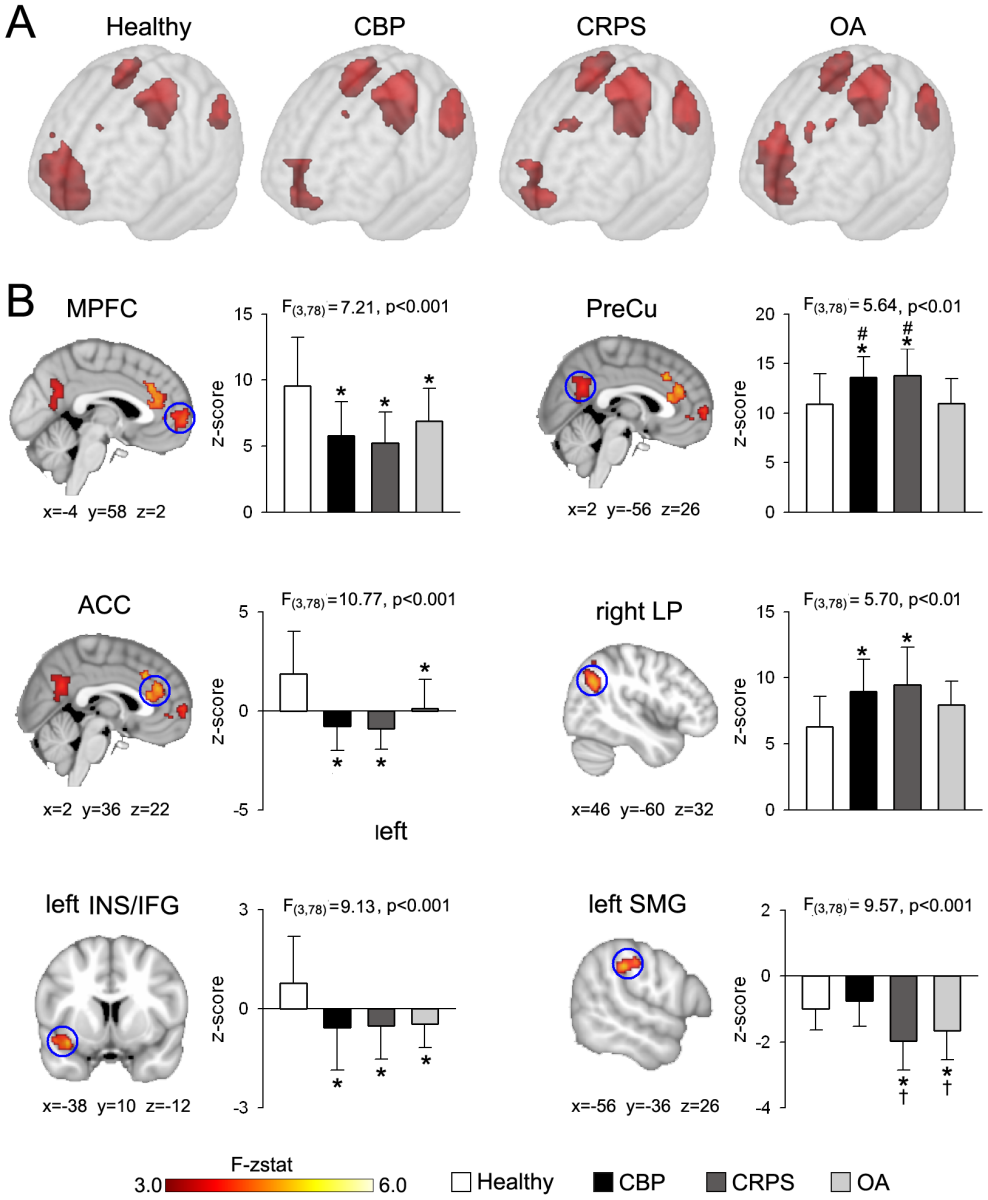
The DMN exhibits divergent connectivity properties across chronic pain patient groups. (**A**) Brain maps show the group average spatial representaion of the DMN for all groups (average map thresholed at z-score >4.0). CBP and CRPS patients show decreased MPFC and increased PreCu and left and right LP representaion within the DMN compared to healthy subjects. (**B**) Maps illustrate clusters of significantly different connectivity for the DMN using a whole-brain voxelwise ANOVA (mixed effects analysis, f-zscore >3.0, corrected for multiple comparisons by cluster threshold p<0.01). All patient groups show decreased connectivity in MPFC (F_3,78_ = 7.21, p<0.001), ACC (F_3,78_ = 10.77, p<0.001), and left anterior INS/IFG (F_3,78_ = 9.13, p<0.001). CBP and CRPS subjects display increased connectivity in PreCu (F_3,78_ = 5.64, p<0.01), compared to healthy controls and OA patients, and in right LP (F_3,78_ = 5.70, p<0.01) compared to healthy controls. In addition, the left SMG exhibits stronger negative connectivity in CRPS and OA groups, than in CBP and control subjects (F_3,78_ = 9.57, p<0.001). Bars represent mean ± S.E.M. of normalized connectivity strength (Post hoc test: *p<0.05 vs healthy; †p<0.05 vs CBP; ‡p<0.05 vs CRPS; #p<0.05 vs OA).

We then examined differences in spatial representation of the DMN across groups. **Figure 2A** shows the group average DMN for the three patient groups and healthy controls. Patients (especially CBP and CRPS) show decreased medial prefrontal cortex (MPFC) and increased precuneus (PreCu) representation within the DMN compared to healthy subjects. Statistical differences in RSN connectivity were computed using each subject's best-fit component images and a ‘whole brain full factorial’ design implemented in FSL to correct for local gray matter density, gender and age (**Figure S1**) following previously described methods [25]. At the subject level, a voxel with a high z-stat value indicates a strong connection to the overall network. Therefore an F-test would reveal which voxels differ in connectivity to the RSN across groups. Out of the five RSNs investigated, only the DMN showed significant local connectivity differences across groups (**Figure 2B** **, Table S2**). Differences in DMN connectivity were localized to brain regions within the network proper including the medial prefrontal cortex (MPFC, F3,78 = 7.21, p<0.001), precuneus (PreCu, F3,78 = 5.64, p<0.01) and right lateral parietal region (LP, F3,78 = 5.70, p<0.01), and to regions outside the DMN such as the anterior cingulate cortex (ACC, F3,78 = 10.77, p<0.001), left anterior insula/inferior-frontal gyrus (INS/IFG, F3,78 = 9.13, p<0.001) and the left supramarginal gyrus (SMG, F3,78 = 9.57, p<0.001).

Post hoc analysis showed that the MPFC, ACC, and left anterior INS/IFG showed similar changes for all patients goups and exhibited stronger positive connectivity to the DMN in controls compared to all patients. On the other hand, the right LP region was more strongly connected to the DMN in CBP and CRPS patients than healthy controls, and showed a borderline differences in OA patients (p = 0.11) The PreCu and the left SMG were the only brain regions that demonstrated group specific differences. The PreCu showed stronger positive correlation to the DMN in CBP and CRPS compared to healthy or OA. On the other hand, the left SMG region showed significant decrease in connectivity for CRPS and OA patients compared to controls or CBP (post hoc comaparisons were considered significant at p<0.05). Thus the DMN exhibited differential connectivity to brain regions within itself and to the rest of the brain. Most of the differences were similar for all patient groups compared to healthy controls, with the PreCu and SMG being the only exceptions. The former showed no change in the OA patients, while the latter was not different in the CBP patients.

In order to assess the effects of the covariates on DMN connectivity, we performed the same analysis without correcting for GM density, age and gender (mixed effects analysis, f-zscore >2.3, corrected for multiple comparisons FWE by cluster threshold p<0.01). Areas that exhibit significant differences in connectivity are similar to those shown in **Figure 2** with the addition of two clusters in the paracentral lobule and intraparietal sulcus (**Figure S2, Table S3**).

### The DMN shows frequency and phase alterations in specific patient groups

Functional connectivity is essentially determined by the frequency content and phase of a signal. In a recent set of studies we showed that the frequency of BOLD oscillations exhibit functional organization in healthy subjects [27] and is altered in chronic pain patients [33]. Here we examined these properties in the DMN to understand how they may contribute to its functional integrity and its interaction with the rest of the brain.

**Figure 3:**
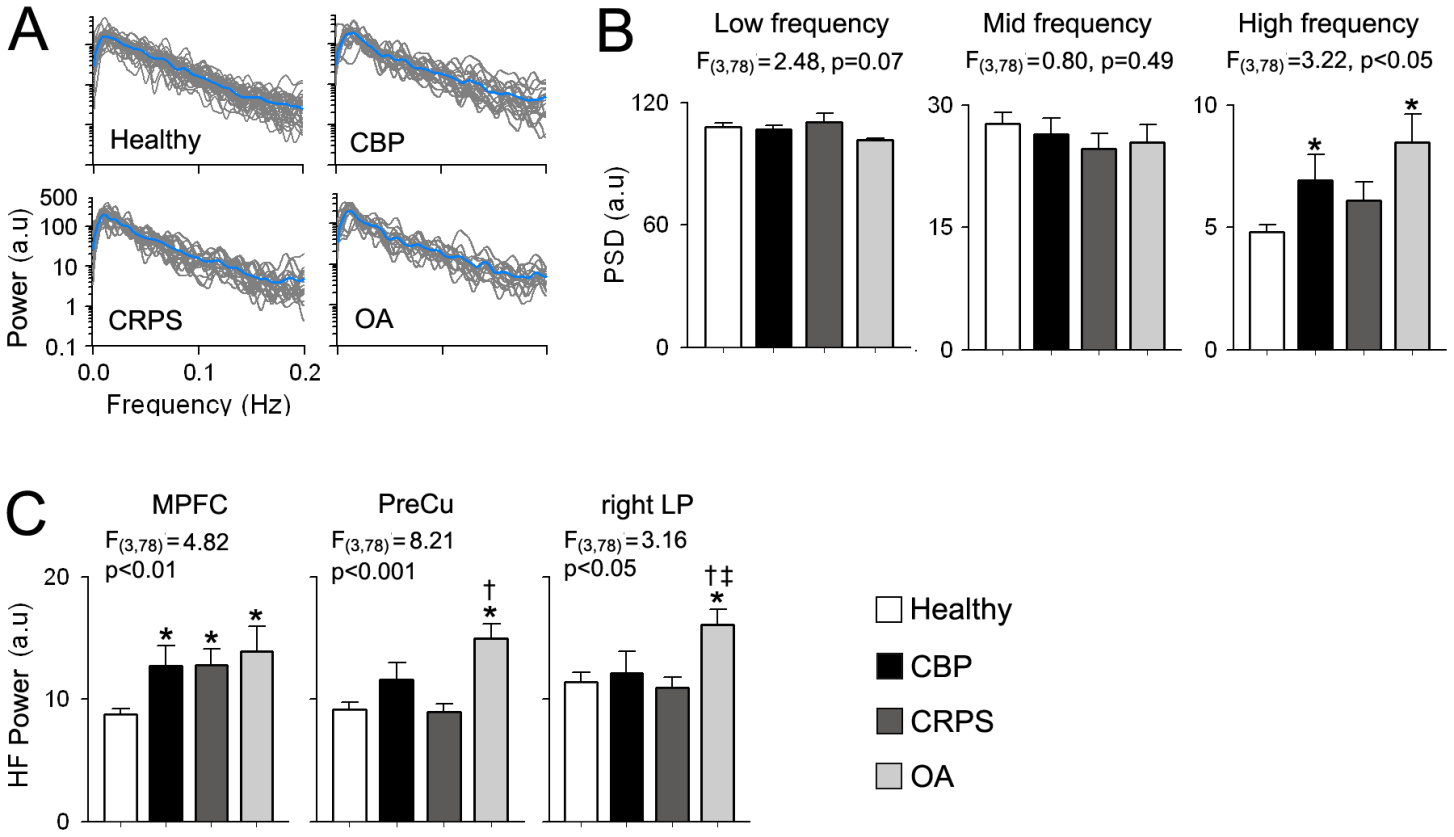
The DMN shows chronic pain type specific increased high frequency oscillations. (**A**) Individual power spectra for the DMN BOLD oscillations superimposed separately for each group. Blue traces represent group averages. (**B**) Bar graphs show the mean ± S.E.M. power from the DMN time courses for the low (0.01–0.05 Hz), mid (0.05–0.12 Hz) and high (0.12–0.2 Hz) frequency bands. CBP and OA patients exhibit increase in power for the high frequency (HF) band compared to controls (F_3,78_ = 3.22, p<0.05, corrected for gender and age). (**C**) Regions within the DMN show differential changes in HF power. All patient groups show increased HF power in MPFC (F_3,78_ = 4.78, p<0.01) compared to healthy controls. On the other hand, only OA patients show increases in HF power in PreCu (F_3,78_ = 8.21, p<0.001) compared to CRPS patients and controls and in right LP (F_3,78_ = 3.16, p<0.05) compared to all groups. (Post hoc test: *p<0.05 vs healthy; †p<0.05 vs CBP; ‡p<0.05 vs CRPS; #p<0.05 vs OA).

**Figure 3A** shows the individual power spectra for the DMN BOLD signal for all groups. We first computed the mean power for 3 frequency bands (low: 0.01–0.05 Hz, mid: 0.05–0.12 Hz and high: 0.12–0.20 Hz) based on previous reports [33], [34] and compared them across groups using an ANCOVA with age and gender modeled as variables of no interest. The DMN exhibited significant group differences in the high frequency (HF) power (F_3,78_ = 3.22, p<0.05), with the CBP and OA groups showing significant increased HF power compared to healthy controls (p<0.05 and p<0.01 respectively), While the OA group showed the largest increase in HF power, it was not significantly different for CBP (p = 0.59) or CRPS (p = 0.09). Differences in oscillatory properties were exclusive to the high frequency band. No differences were detected for the low (F_3,78_ = 2.84, p = 0.07) and mid frequency (F_3,78_ = 0.80, p = 0.49) bands. (**Figure 3B**). We also computed the HF power for the three regions within the DMN (MPFC, PreCu and right LP) that exhibited connectivity differences as shown in **Figure 2B**. All three regions showed significant across-group differences in HF power: MPFC (F_3,78_ = 4.82, p<0.01), PreCu (F_3,78_ = 8.21, p<0.001) and right LP (F_3,78_ = 3.16, p<0.05). While the MPFC exhibited increased HF power for CBP (p<0.05), OA (p<0.01) and CRPS (p<0.05), the PreCu and LP showed differential changes across pain groups. The PreCu showed increased HF power only in OA patients compared to healthy controls and CRPS patients (p<0.01 for both regions and groups), while LP showed increased HF power in OA that was significantly different from all groups (p<0.05) (**Figure 3C**).

**Figure 4:**
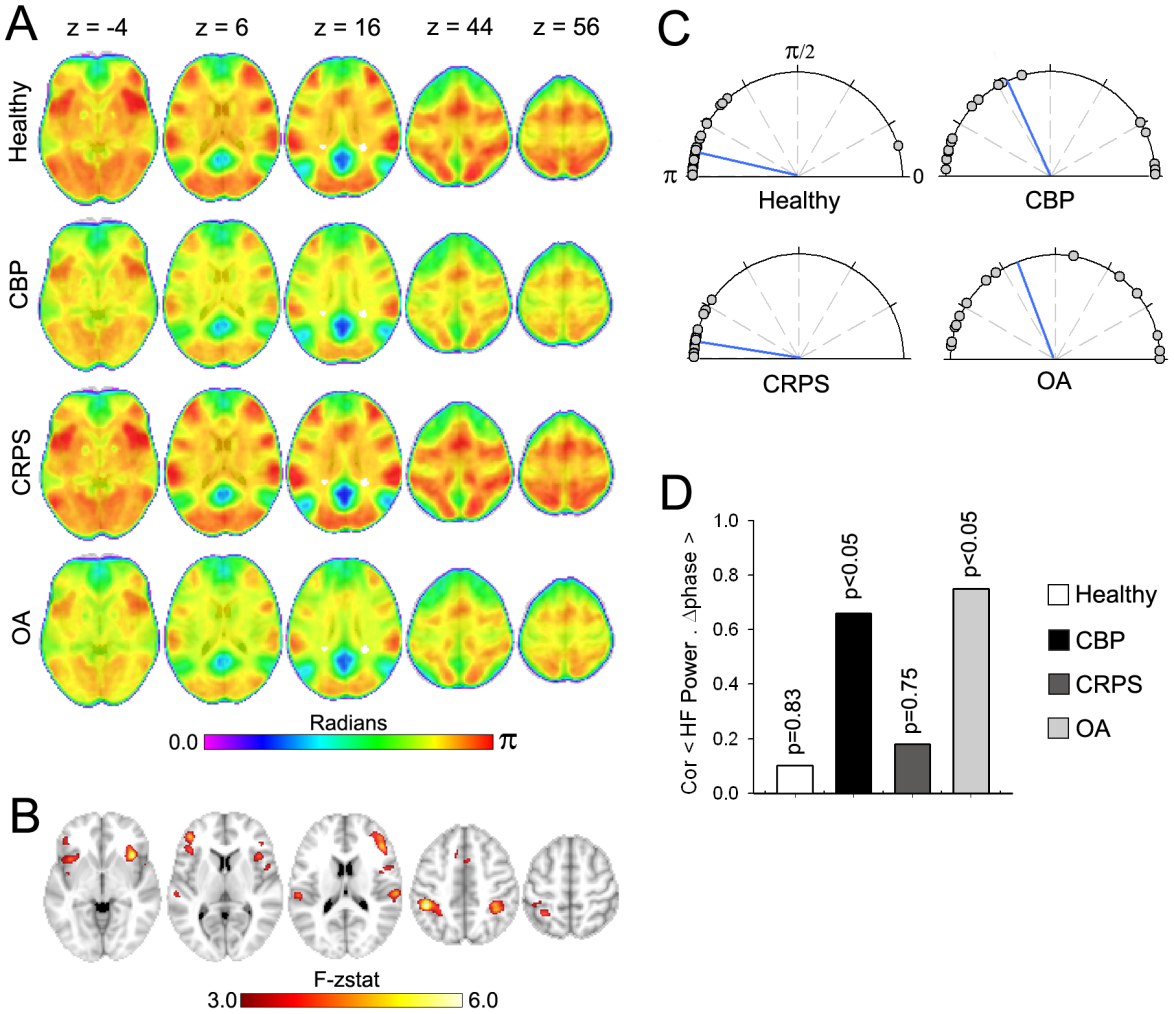
The DMN shows chronic pain type specific changes in phase properties. (**A**) Brain maps show the group voxelwise average phase differences (Δphase) between the DMN time course and all other brain voxels. Blue-green areas represent smaller phase differences while yellow-red represents greater phase differences. In general CBP and OA patients exhibited decreased phase differences, compared to healthy subjects and CRPS patients. (**B**) Brain map illustrates clusters of significantly different phase relationship to the DMN, using a whole-brain voxelwise ANOVA (mixed effects analysis, f-zscore >3.0, corrected for multiple comparisons by cluster threshold p<0.01). The DMN in patients show changes in phase relationships to regions within the frontoparietal network inculding bilateral IPS, and FEF in addition to the right DLPFC, and to regions within the salience network including ACC and bilateral anterior and posterior insula. (**C**) Compass plots show the individual absolute phase differences (Δphase) between the DMN and the network identified in **B** for all groups. Watson-Williams test for circular data reveals a significant difference of mean phase across groups (F_3,78_ = 7.45, p<0.01). Blue lines represent the circular mean. (**D**) Correlation between Δphase and DMN HF Power. Only CBP and OA patients show a significant relationship.

Next we investigated phase properties of the DMN. First we performed a Hilbert transformation for every voxel in the brain to extract the phase-time series and subtracted it from the phase-time series of the DMN. The resultant phase difference (Δphase) was averaged across time to obtain the mean phase difference to the DMN for every voxel. Mean group Δphase maps for all groups are shown in **Figure 4A**. In general voxels within the DMN exhibited small Δphase values across all groups (blue-green regions), while frontal and parietal regions showed larger Δphase (yellow-red regions) in healthy and CRPS patients compared to CBP and OA patients. A whole brain voxel-wise Watson-Williams F-test was then performed to determine voxels that differed in phase relationship to the DMN across groups (mixed effects, z>3.0, corrected for multiple comparisions by cluster threshold p<0.01). Regions showing phase differences with the DMN are shown in **Figure 4B** and mainly ovelapped with the frontoparietal network (spatial conjunction = 53.78%) and salience network (spatial conjunction = 21.34%). Local foci and their respective coordinates are listed in **Table S3** and included bilateral intraparietal sulcus (IPS) and INS, in addition to middle cingulate cortex, right frontal eye field and dorsolateral prefrontal cortex (DLPFC). Phase differences for individual subjects between the DMN time course and the network identified above are displayed in **Figure 4B**. While CRPS and control subjects exhibited phase differences around π radians (i.e anti-correlation), CBP and OA patients showed more varied relationship with an average phase closer to π/2 radians. A Watson-Williams F-test for circular data revealed a significant difference of phase across groups (F_3,78_ = 7.45, p<0.01), with the OA and CBP patients showing significantly higher coupling than CRPS patients and healthy controls (p<0.01 for all comparisons).

We then examined the relationship between DMN phase and frequency. We observed that increased power in the HF band was strongly correlated to Δphase in the CBP (R = 0.62, p<0.05) and OA (R = 0.73, p<0.05), but not in CRPS (R = 0.19, p = 0.75) or healthy controls (R = 0.09, p = 0.83) (**Figure 4D**).

### DMN frequency and phase changes are related to pain duration in specific patient groups

**Figure 5:**
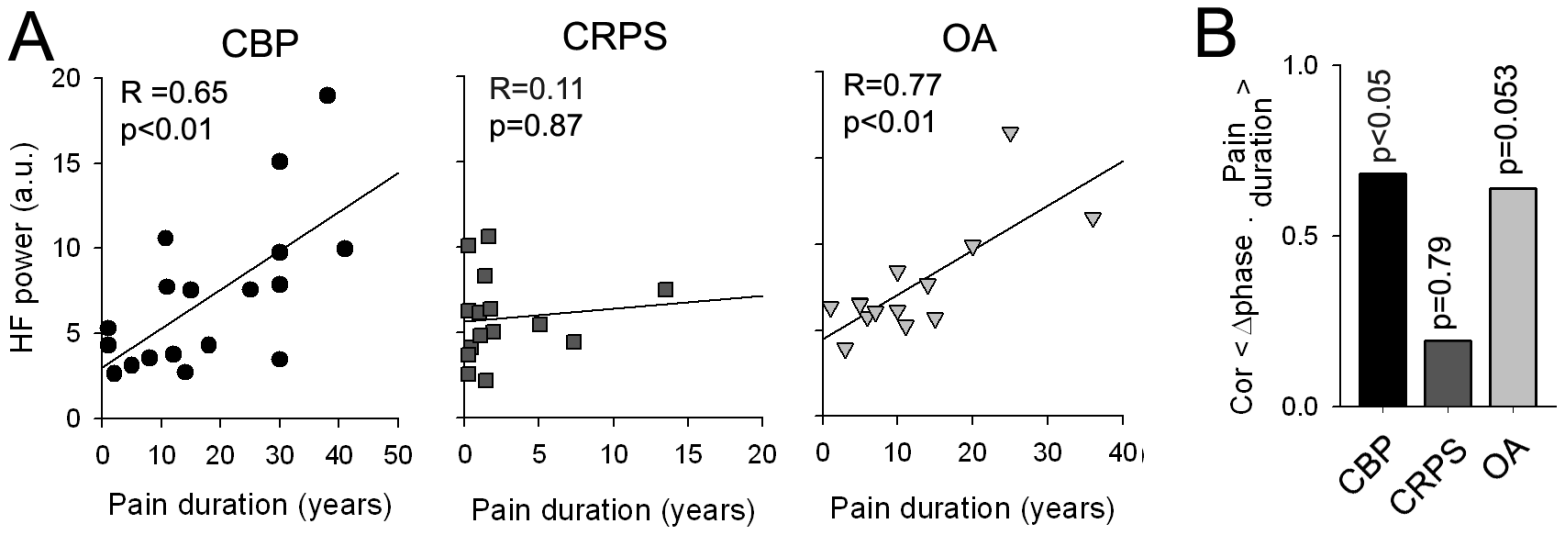
DMN spectral power and phase changes are related to pain duration in specific patient groups. (**A**) The DMN high frequency spectral power shows significant positive correlation to pain duration in CBP (R = 0.65, p<0.01) and OA (R = 0.77, p<0.01), but not in CRPS (R = 0.11, p = 0.87). (**B**) Phase differences between the DMN and frontoparietal network shows high correlation to pain duration in CBP (R = 0.68, p<0.05), a positive trend in OA (R = 0.64, p = 0.053) and no correlation in CRPS (R = 0.19, p = 0.79). Note pain duration is significanlty less in CRPS, than in CBP (t-value  = −4.56, p<0.01) and OA (t-value  = −3.34, p<0.01).

We investigated the association of the observed DMN frequency and phase changes with the clinical characteristics of patient groups, namely pain duration and intensity. Association of HF power with pain parameters was computed for each group separately using a Pearson correlation between individual HF power and pain parameters. HF power within the DMN showed a strong positive correlation with pain duration in the CBP (R = 0.65, p<0.01) and OA (R = 0.77, p<0.01) groups, but not in CRPS (R = 0.11, p = 0.87) (**Figure 5A**). Phase relationship with pain parameters was computed using a circular correlation between Δphase and pain parameters. Similar to frequency results, phase differences between the DMN and frontoparietal network were also significantly positively correlated to pain duration in CBP (R = 0.68, p<0.05), showed a positive trend in OA (R = 0.64, p = 0.053), and was not significant in CRPS (R = 0.19, p = 0.79) (**Figure 5B**). Neither phase nor frequency was related to pain intensity in any group (**Table S4**).

We also investigated whether the DMN size, which was significantly different across groups, showed any correlation with pain duration and intensity. Neither duration nor intensity showed significant correlations with DMN size for any patient group (**Table S4**). It is important to note that we also investigated the correlation of HF power and Δphase with depression and drug usage. Neither parameter showed a significant correlation to duration or intensity of pain (all p-values >0.05).

### The MPFC connectivity changes are related to pain intensity at the time of the scan in patients

Given that the MPFC showed significant connectivity and frequency differences across all patient groups, we further examine its functional properties in order to elucidate the mechanisms underlying these changes.

**Figure 6:**
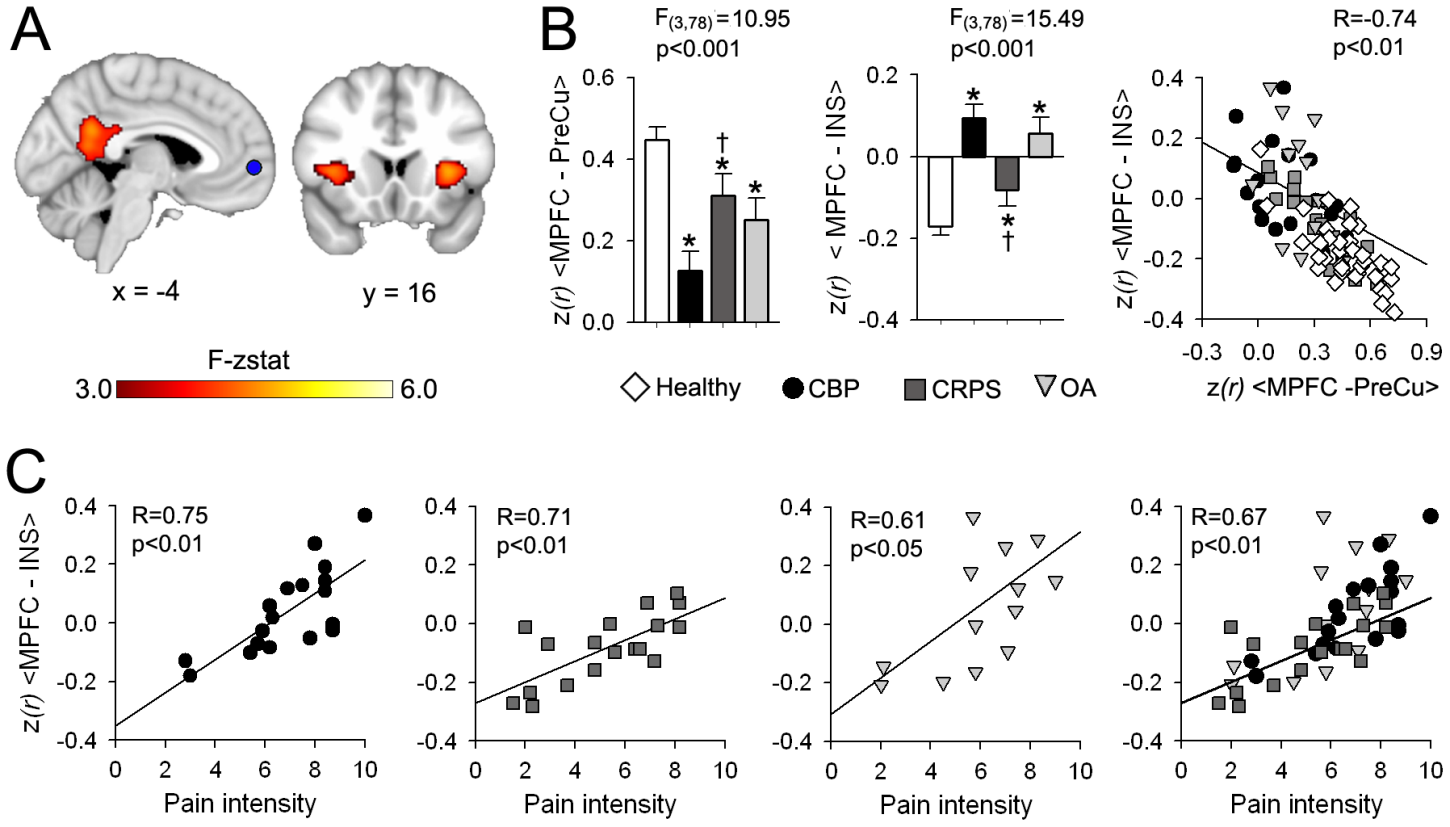
MPFC exhibits connectivity changes in proportion to intensity of pain. (**A**) Brain map illustrates regions showing significantly different correlation to the MPFC across all groups using a whole-brain voxelwise ANCOVA corrected for age and gender(mixed effects analysis, f-zscore >3.0, corrected for multiple comparisons by cluster threshold p<0.01). Differences in MPFC connectivity between groups were restricted to the bilateral anterior INS and PreCu. (**B**) Bar graphs show the mean ± S.E.M. normalized correlation (z*(r)*) for MPFC-PreCu and MPFC-INS for all groups. All patients show significant decrease in MPFC-PreCu corelletion (F_3,78_ = 7.18, p<0.001, corrected for gender and age) and increase in MPFC-INS correlation (F_3,78_ = 8.38, p<0.001). In addition, CBP patients showed lower MPFC-PreCu and higher MPFC-INS compared to CRPS patients. Right scatter plot shows the relationship between MPFC-INS and MPFC-PreCu association. Increase in the MPFC-INS correlation was inversly related to MPFC-DMN connectivity across all subjects (R = −0.74, p<01). (**C**) MPFC-INS connectivity showed high correlation to pain intesity in CBP (R = 0.75, p<0.01), CRPS (R = 0.71, p<0.01) and OA (R = 0.61, p<0.05). This correlation was maintaintended when examined across all patient groups (R = 0.67, p<0.01). (Post hoc test: *p<0.05 vs healthy; †p<0.05 vs CBP).

Changes in MPFC connectivity was quantified using a whole brain intrinsic correlation analysis. For each subject the MPFC BOLD timecourse was extracted and its correlations to all voxels to the brain was computed using previously described methods [29], [30]. Group differences in MPFC correlation to the rest of the brain was assessed using a whole brain voxel-wise ANCOVA, with age and gender as covariates of no interest (**Figure 6A**). The MPFC showed significant differences in correlation strength to bilateral INS (F_3,78_ = 8.38, p<0.001) and PreCu (F_3,78_ = 7.18, p<0.001). The mean ± S.E.M. of MPFC-PreCu (correlation between MPFC and PreCu) and MPFC-INS (correlation between MPFC and INS) are shown in **Figure 6B**. Post-hoc analysis showed that all patients exhibited decreased MPFC-PreCu connectivity and increased MPFC-INS connectivity, compared to controls (p<0.05 for all contrasts). CBP pateints showed the most robust MPFC connectivity changes among the three patient population. MPFC-PreCu connectivity was signifcantly lower in CBP compared to CRPS (p<0.05), while the MPFC-INS connectivity was significantly higher for the same comparison (p<0.05).

Next we examined the relationship between MPFC–INS and MPFC-PreCu across all subjects. Results are displayed in the scatter plot in **Figure 6B**. the MPFC–INS and MPFC-PreCu exhibited a significant negative correlation (R = −0.74, p<0.01).

Finally, We explored whether the MPFC functional connectivity changes (especially MPFC-INS connectivity) were related to pain parameters. We found that the MPFC-INS connectivity was significantly correlated to pain intensity at the time of the scan in CBP (R = 0.75, p<0.01), CRPS (R = 0.71, p<0.01) and OA (R = 0.61, p<0.05). This correlation was maintainted when examined across all patient (R = 0.67, p<0.01) (**Figure 6C**).

## Discussion

Recent neurobiological theories [35], [36] have emphasized the importance of the interaction between brain regions in explaining higher brain function. Here we report synchronization changes in the default mode network across three chronic pain patient populations with distinct clinical etiologies. These changes were found at rest (i.e. in the absence of overt stimulation) and thus reflect cortical functional reorganization in the presence of spontaneous pain (all patients were experiencing pain during the resting state fMRI scans). In addition to connectivity changes, the DMN showed increased high frequency BOLD oscillations (0.12–0.20 Hz) within the MPFC for all patient groups, but also in preCu and right LP in OA. The DMN also showed changes in phase relationship with nodes of the frontoparietal network, which encompasses brain regions involved in attention and working memory, and with multiple regions of the insula and with ACC implying modulation by pain. Furthermore both phase and frequency changes were related to pain duration. The decrease in connectivity between MPFC and PreCu was 1) observed in all three patient groups, 2) was inversely proportional with increased connectivity between MPFC and bilateral insula, and 3) the strength of MPFC and insula connectivity reflected pain intensity in all three chronic pain patient groups. These findings highlight the complexity of neural mechanisms underlying chronic pain and suggest that continued living with chronic pain distrorts the interplay amongst multiple brain networks.

Resting state networks are an intrinsic property of the brain since they can be found across various behavioral and physiological states, including sleep [37] anesthesia [38], and task performance [39]. Resting state networks have been shown to exhibit functional specificity and have been linked to known anatomical systems [31], [39], [40]. The DMN remains the most extensively studied and characterized resting state network. It is found to be more ‘active’ when the subject is at rest, i.e. deactivating in subjects responding to stimuli, and is modulated by task difficulty [41]. Recent functional imaging studies in humans show the DMN to be disrupted in central disease states such as Alzheimer's disease, autism and schizophrenia (for review see [42]), and modulated by the presence of chronic pain in different clinical pain conditions, see review [7]. However the extent of these changes, and to what degree the DMN reorganization is specific/common across chronic pain conditions has remained largely unknown.

Here we show that the DMN exhibits common changes in synchrony within its elements and the rest of the brain in all three patient cohorts tested compared to healthy controls. These changes can be generalized in two seminal observations: 1) Increased correlation of the DMN, specifically the MPFC with insular cortex and 2) decreased connectivity of MPFC with posterior constituents of the DMN, especially the PreCu. Increased connectivity between the insula and the MPFC has been observed in other studies. Tagliazucchi and colleagues showed that brain regions within the frontal cortex, mainly orbital areas, exhibit increased co-activation with bilateral insula in CBP patients during resting state fMRI, but not in healthy controls [5]. Increased association of the DMN and insula was also observed in patients suffering from diabetic neuropathy [43], fibromyalgia [6] and temporomandibular disorder [10] compared to healthy controls. More importantly the functional correlation of insular regions with portions of the MPFC was related to clinical pain parameters: it decreased after successful treatment of pain in fibromyalgia [44] and increased after exacerbating pain in chronic back pain patients [9]. Consistent with these results and complementing them, the present analysis shows that insula and MPFC connectivity in all three patient groups correlates with the intensity of the respective pain conditions. The insular cortex shows a very high incidence of activation in pain tasks [17] and parts of the region are considered to signal either sensory or emotional/affective properties of pain [16], and differentiate between encoding of nociceptive information or subjective pain perception [45]. Thus our results suggest that the DMN shows increased coupling with pain-related regions altering brain dynamics at rest. Our results also show decreased MPFC connectivity with the PreCu in all patient groups compared to healthy controls. The PreCu has been shown to be involved in autobiographical and episodic memory retrieval and mentalizing. It is primarily involved in elaborating and integrating information rather than directly processing stimuli [46], [47], [48]. This decreased functional connectivity of the MPFC with other parts of the DMN was directly related to its increase with insula, thus suggesting that chronic pain might modulate higher cognitive processes by altering normative functions of the DMN, as a direct consequence of pain interfering with the MPFC function within the DMN. These observations suggest that the MPFC is losing its “membership” within the DMN in patients compared to controls, which is caused by its increased connectivity to anterior INS, a brain region known to be consistly involved in encoding nociceptive information of painful stimuli [1], [49].

These synchrony changes within the DMN were also associated with changes in oscillatory properties of BOLD signal, namely the frequency and phase. The DMN or parts of the DMN exhibited an increased oscillation shift toward the high frequency domain. We observed that all patients showed a shift towards higher frequencies in the MPFC, thus corroborating our previous findings [33] in CBP patients, as well as reports from two other patient populations [8], [34]. The functional relevance of high frequency BOLD oscillations (0.10 to 0.25 Hz) is not fully understood. However there is accumulating evidence showing that changes in BOLD oscillation is related to changes in behavioral state such as task performance and thus may reflect changes in neural activity [27], [50]. Thus the increases in high frequency power in the DMN could reflect changes in ongoing neural activity and/or cortical excitability associated with spontaneous pain. We recently demonstrated that MPFC high frequency oscillations reflect spontaneous pain in CBP patients [33], whether this generalizes to other chronic pain conditions remains to be shown.

Multiple studies have shown that the the DMN may work in direct opposition to other systems, especially with those invovled in external attention [30], [51], [52], [53]. In this study, the phase analysis systematically and quantitatively identifies this relationship between the DMN and other brain voxels. In the healthy subjects (and in CRPS), consistent with the literature, we observe that most brain voxels are about π radians from DMN. In CBP and OA, however, average phase between DMN and the rest of the brain is closer to π/2, that is the DMN is shifting from anti-correlation to an orthogonal state, rendering the inhibitory/competitive interaction between DMN and the rest of the brain to become less interdependent. Specifically, the decoupling of the DMN was with the frontoparietal network, which is shown to be primarily involved in attention. This result replicates our previous finding showing decreased anti-correlation between the DMN and the attention network in CBP during task performance [29]. In addition, we observe decouping for multiple parts of the insula and also the ACC (regions involved in nociception and pain), which suggests that the changes in phase locking can be thought of as compensation of resources, and interaction between pain and attention.

It should be noted that the two main characteristics of chronic pain, intensity and duration, both influence the reorganization of the DMN. Increased high frequency power and phase shift were inter-related, and different mainly in CBP and OA, but not for CRPS. As figure 5 illustrates, the main difference between the CRPS group and OA and CBP groups was the mean duration of pain persistence. It seems that for pain durations less than 10–15 years, high frequecny power (and also probably phase) is minimally disrupted in all three types of chronic pain. On the other hand, pain intensity seems to modulate the DMN independently from its duration as it impacts the connectivity between MPFC and insula similarly for all three patient groups, regardless of pain duration differences between the groups.

We recently demonstrated that the strength of connectivity between MPFC and nucleus accumbens causally predicts transition to CBP at one year prior [54], implying that the strength with which MPFC modulates mesolimbic learning circuitry determines the extent of vulnerability of a given subject to developing chronic pain following an acute/sub-acute pain episode. Here we observe, in all three patient groups, that MPFC membership to DMN is decreased, its high frequency oscillations are increased in proportion to pain duration, and its connectivity to the insula is increased in proportion to intensity of chronic pain. Thus, the MPFC must be considered a common and critical node within the circuitry underlying chronic pain as it links the reorganization of the DMN with that of the brain mesolimbic learning circuitry. The earliest cortical reorganization observed in the transition from acute/sub-acute back pain to chronic pain is the functional connectivity of the insula [54]. We speculate that this reorganization in time translates into increased information sharing between the insula and MPFC reflecting the transition of pain from a sensory and nociceptive state to becoming more of an emotional burden which then disrupts DMN properties. Mechanisms that link these cortical and sub-cortical reorganization with peripheral and spinal cord plasticity remains to be studied.

We have previously shown that cortical gray matter denisty is distinct between the three groups of chronic pain patients studied here [18]. To decrease this confound we covaried gray matter density for each group [25]. This approach enabled identifying DMN characteristics that are changed commonly across all three groups. However, even after this correction we also observe changes in the DMN that seem specific to each type of chronic pain. These specific changes entail mainly spatial extent of components of the DMN as well as the stength of connectivity within each of the components comprising the DMN (in addition to high frequency power increase in PreCu and right LP in OA patients). For example, the overall size of the DMN was larger in CBP and CRPS, but not in OA; the within cluster connectivity strengths were higher in PreCu and right LP in CBP and CRPS, but not in OA; while in left SMG stronger negative internal connectivity was seen in CRPS and OA, but not in CBP. These spatial changes in DMN were not influenced by pain intensity or duration. Instead we surmise they reflect more complex cognitive/emotional suffering, coping, and learning characteristics associated with the distinct chronic pain conditions.

One major concern in the study is that head motion artifacts are aliased into the BOLD signal especially in the high frequency spectrum that showed differences between patients and controls [32], [50]. We examine and compare head motion displacement in all groups to ensure that differences in functional connectivity and frequency content between patients and healthy subjects are not artifactual. Mean head motion in healthy subjects and patients during resting state fMRI are shown in Figure S3. All groups showed minimal head displacement and there were no differences across groups when compared using an ANOVA. Furthermore, motion did not show any significant correlation with all functional properties measured, including functional connectivity, frequency, phase across all groups (**Table S5**). An additional limitation of the study is the fact that chronic pain patients use various analgesic drugs over many years, which might confound observed brain functional and morphological changes. We quantified drug consumption using a validated questionnaire [21], which reduces drugs used for different durations and doses to a single scalar. This allowed us to examine the effect of medication on various functional parameters using a covariate analysis. Minimal relationships were observed (**Table S6**). It is important to note that different kinds of medication with different mechanisms of action might exert unique effects on resting state brain networks. For example, it has been shown that ketamine (an anti-depressant drug) decreases functional connectivity of the DMN to the MPFC in healthy subjects [55]. Here we do not control for the frequency of intake of different classes of medication in the different chronic pain conditions. This is mainly due to the relatively small number of patients and the lack of a proper control. Thus medication intake remains the biggest confound in this study, and an important issue for future clinical pain research. Since chronic pain and depression have high comorbidity, we investigated the Relationship between depression and all functional paramters assessed in the study. Overall, depression showed low correaltions with all functional properties measured (**Table S7**).

In conclusion, we show that various types of clinical chronic pain conditions are associated with functional connectivity changes within the DMN during resting state fMRI. The reorganization common between patient groups is the extent of association of the medial prefrontal component of the DMN with the insula, and its dissociation from the posterior components of the DMN, which seems to disrupt the competitive inhibition between the DMN and the brain networks underlying attention. The extent of this reorganization is a function of the intensity of the chronic pain and the duration of its persistence, with some of these changes occuring mainly after more than a decade living with chronic pain. On the other hand, reorganization of spatial properties of the DMN seems more specific to each type of chronic pain, which may reflect different emotional, attentional, and cognitive abnormailites observed in various clinical chronic pain conditions.

## Supporting Information

Figure S1 **ICA comparison design.** (A) Brain slices represent the five resting state networks examined in the study. Shown are the templates used for identifing each subjects' best fit component. The templates were generated using a group independent component analysis on an independent data set from healthy controls (Baria et al., 2011). We selected five components that coincided with resting state networks described in previous studies (Damoiseaux et al., 2006; De Luca et al., 2006) and including the default mode, salience, sensorimotor, frontoparietal and visual netowrks. (B) Study design schematic used for comparison of group differences in independent component analysis shown in Figure 2. Preprocessed task-free fMRI data were decomposed using independent component analysis, and were identified for each subject by calculating goodness-of-fit to templates shown in (A). Grey matter maps were also derived from T1-weighted structural MRI data of each subject for atrophy correction. Differences between-group connectivity alterations were tested for all networks using a whole brain voxel-wise ANCOVA with GM density and age as contious variable, and gender and group as catagorical values. Statistical maps were corrected for multiple comparison using the fsl cluster correction (p<0.01) which utilizes gussian random field theory. CBP  =  chronic back pain; CRPS  =  complex regional pain syndrom; OA  =  osteoarthritis; ICA  =  independent component analysis; GM  =  gray matter (Flow chart adapted for Zhou et., 2011).
(TIF)

Figure S2 **Group differences in DMN ICA analysis without GM correction.** Maps illustrate clusters of significantly different connectivity for the default mode network (DMN) using a whole-brain voxelwise ANCOVA without correcting for GM density, age and gender (mixed effects analysis, f-zscore >2.3, corrected for multiple comparisons by cluster threshold p<0.01). Areas that exhibit significant differences in connectivity are similar to those shown in Figure 2 with the addition of 2 clusters in the paracentral lobule (PCL) and intraparietal sulcus (IPS). List of regions and corresponing coordinates are presented in Table S3.
(TIF)

Figure S3 **Assesment of head motion artifacts in healthy subjects and patients during RSN.** (A) Time series plots depict absolute head displacement during functional scans which is estimated from the three translational and three rotational parameters obtained by rigid body correction of head motion. Head displacement relative to its position mid way through the scan (t = 300 seconds) is routinely computed (and corrected) in each subject by the MCFLIRT program, part of FSL software package. Additionally, head motion time courses are also used in all first level analyses as a covariate of no interest (see methods for details), as a second step to further minimize its contribution to brain activity. The plot depicts the group average head motion as a function of time (lines correspond to the mean values and bars are standard errors, plotted every 25 seconds), in general deviations are smaller than 2 mm (smaller than the voxel size) during all functional scans. (B) Bars represent the group average mean absolute displacement ± SEM for each group. The average mean absolute displacemnt is computed for each subject seperately by averaging all head displacement in time. There were no significant differences.
(TIF)

Table S1 **Demographic and clinical data for CBP, CRPS and OA patients that participated in the fMRI study.** BDI  =  Beck's depression inventory; M =  male; F =  female; VAS = visual analogue scale. The VAS was computed from the McGill short-form questionnaire (sf-MPQ). MQS is medication quantification questionnaire.
(DOCX)

Table S2 **Coordinates for peak foci for DMN ICA analysis.** All coordinates listed in MNI space x, y, z values in mm.; MPFC  =  medial prefrontal cortex; PreCu  =  Precuneus; ACC  =  anterior cingulate cortex; LP  =  lateral parietal; IFG  =  Inferior frontal gyrus; INS  =  insula; SMG  =  supramarginal gyrus.
(DOCX)

Table S3 **Coordinates for peak foci for DMN ICA analysis without Gray matter correction.** All coordinates listed in MNI space x, y, z values in mm; MPFC  =  medial prefrontal cortex; PreCu  =  Precuneus; ACC  =  anterior cingulate cortex; LP  =  lateral parietal; IFG  =  Inferior frontal gyrus; INS  =  insula; SMG  =  supramarginal gyrus; PCL  =  paracentral lobule; IPS  =  intra parietal sulcus.
(DOCX)

Table S4 **Relationship between DMN properties and clinical parameters in patients.** Correlation between pain intensity or duration with DMN size, high frequency (HF) power and Δphase for all groups of patients computed seperately (data from Figure 4) [* p<0.05, ** p<0.01].
(DOCX)

Table S5 **Relationship between mean absolute displacement and all functional paramters assessed in the study.** There was no signifecent correlation with head motion and functional connectivity parameters across all groups.
(DOCX)

Table S6 **Relationship between Drug usage and all functional paramters assessed in the study.** Except for DMN-left LP correlation in CBP and sensorimotor size in CRPS, there was no signifecent association between drug usage and all functional parameters assessed in the study. (significent relationships are shown in red). MQS is a validated pain medication use questionnaire, which generates equivalences between various analgesic drugs.
(DOCX)

Table S7 **Relationship between depression and all functional paramters assessed in the study.** Except for left SMG connectvity and sensorimotor size in OA, there was no signifecent association between depression, measured used the Beck's deprresion index, and all functional parameters assessed in the study. (significent relationships are shown in red).
(DOCX)
