# Supplementary figures and images for: Functional Reorganization of the Default Mode Network across Chronic Pain Conditions
Source: PLoS One. 2014 Sep 2;9(9):e106133. doi: 10.1371/journal.pone.0106133 (PMC4152156; doi:10.1371/journal.pone.0106133)

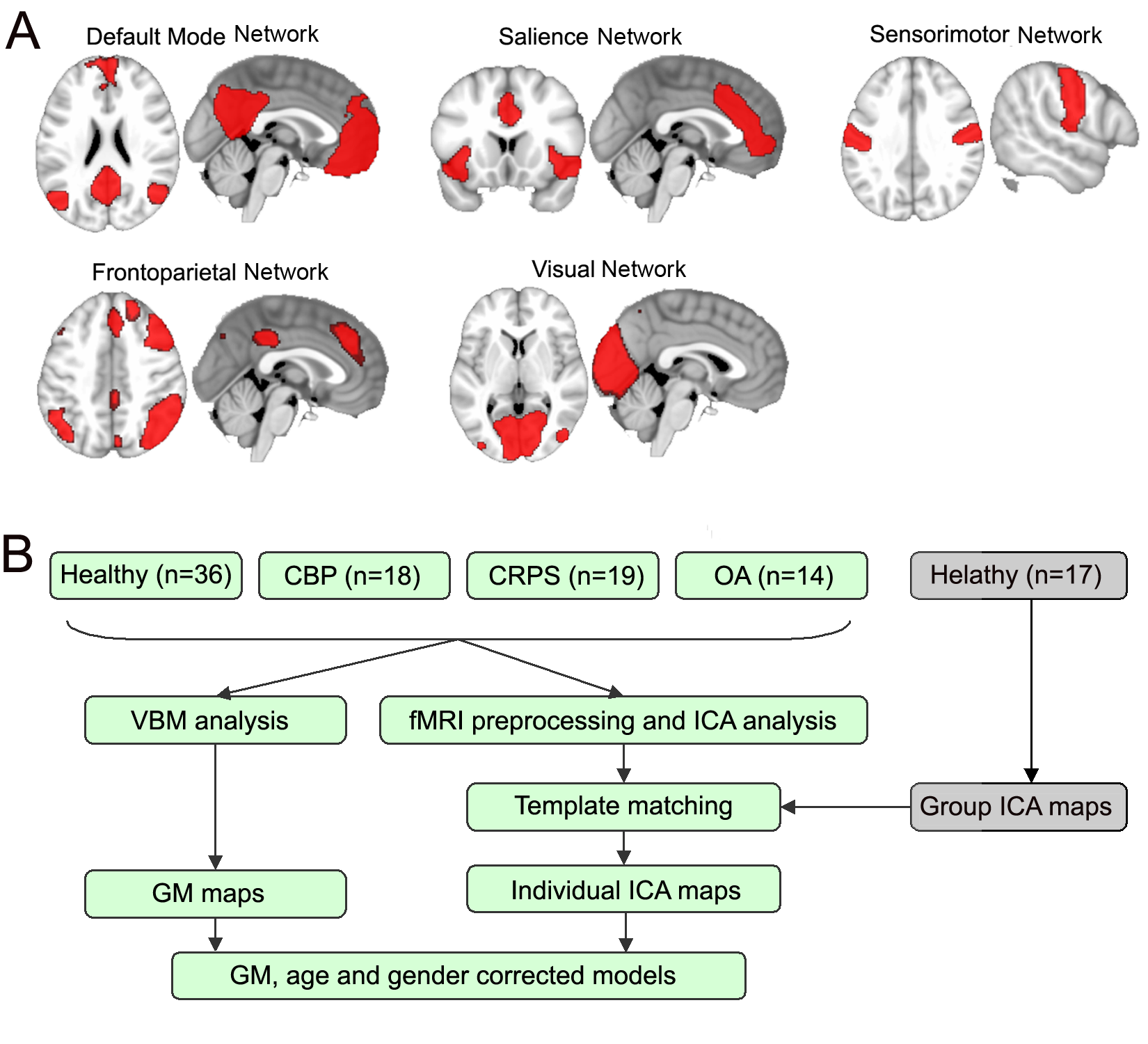

Supplement: Figure S1 — ICA comparison design. (A) Brain slices represent the five resting state networks examined in the study. Shown are the templates used for identifing each subjects' best fit component. The templates were generated using a group independent component analysis on an independent data set from healthy controls (Baria et al., 2011). We selected five components that coincided with resting state networks described in previous studies (Damoiseaux et al., 2006; De Luca et al., 2006) and including the default mode, salience, sensorimotor, frontoparietal and visual netowrks. (B) Study design schematic used for comparison of group differences in independent component analysis shown in Figure 2. Preprocessed task-free fMRI data were decomposed using independent component analysis, and were identified for each subject by calculating goodness-of-fit to templates shown in (A). Grey matter maps were also derived from T1-weighted structural MRI data of each subject for atrophy correction. Differences between-group connectivity alterations were tested for all networks using a whole brain voxel-wise ANCOVA with GM density and age as contious variable, and gender and group as catagorical values. Statistical maps were corrected for multiple comparison using the fsl cluster correction (p<0.01) which utilizes gussian random field theory. CBP = chronic back pain; CRPS = complex regional pain syndrom; OA = osteoarthritis; ICA = independent component analysis; GM = gray matter (Flow chart adapted for Zhou et., 2011). (TIF) [file pone.0106133.s001.tif]

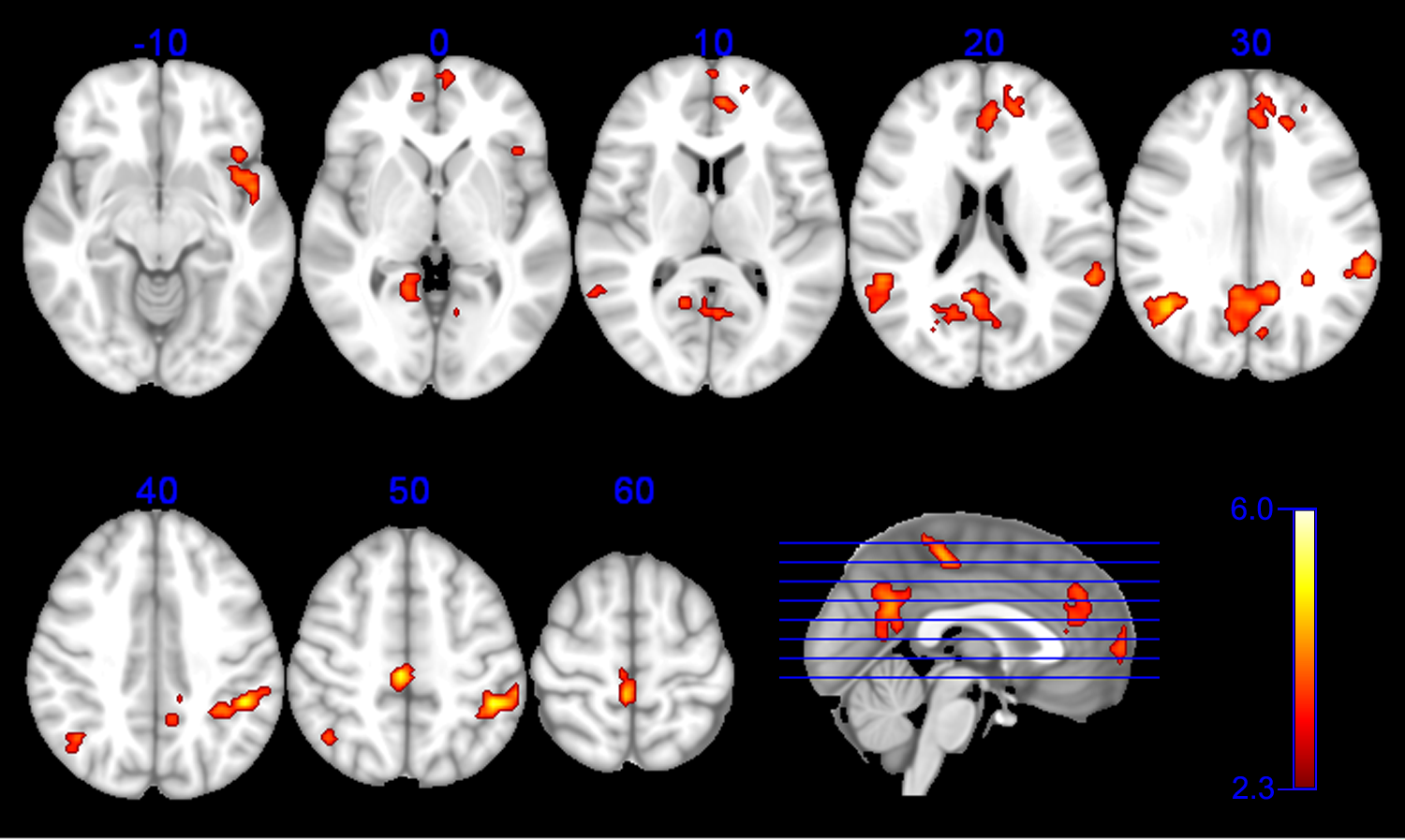

Supplement: Figure S2 — Group differences in DMN ICA analysis without GM correction. Maps illustrate clusters of significantly different connectivity for the default mode network (DMN) using a whole-brain voxelwise ANCOVA without correcting for GM density, age and gender (mixed effects analysis, f-zscore >2.3, corrected for multiple comparisons by cluster threshold p<0.01). Areas that exhibit significant differences in connectivity are similar to those shown in Figure 2 with the addition of 2 clusters in the paracentral lobule (PCL) and intraparietal sulcus (IPS). List of regions and corresponing coordinates are presented in Table S3. (TIF) [file pone.0106133.s002.tif]

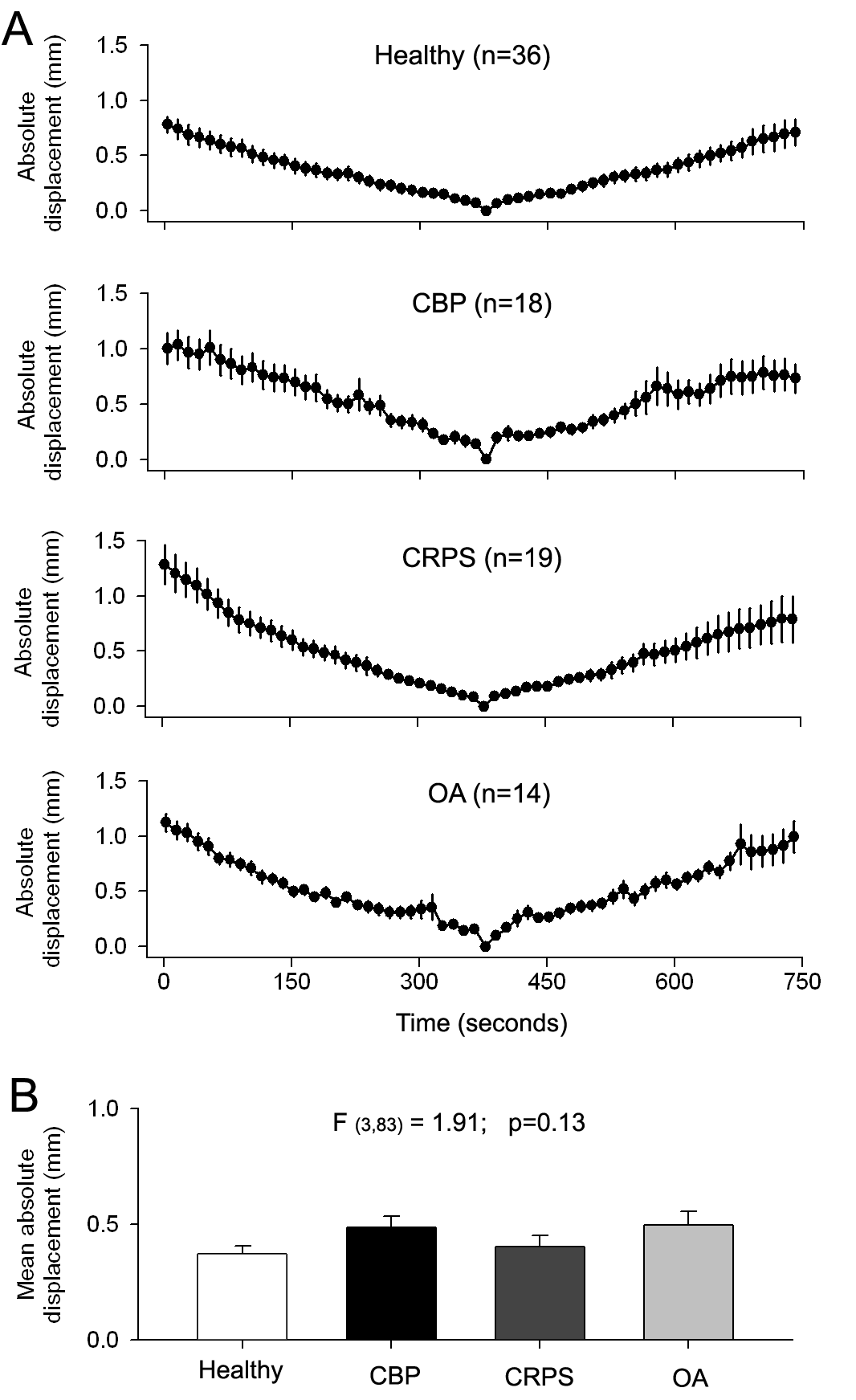

Supplement: Figure S3 — Assesment of head motion artifacts in healthy subjects and patients during RSN. (A) Time series plots depict absolute head displacement during functional scans which is estimated from the three translational and three rotational parameters obtained by rigid body correction of head motion. Head displacement relative to its position mid way through the scan (t = 300 seconds) is routinely computed (and corrected) in each subject by the MCFLIRT program, part of FSL software package. Additionally, head motion time courses are also used in all first level analyses as a covariate of no interest (see methods for details), as a second step to further minimize its contribution to brain activity. The plot depicts the group average head motion as a function of time (lines correspond to the mean values and bars are standard errors, plotted every 25 seconds), in general deviations are smaller than 2 mm (smaller than the voxel size) during all functional scans. (B) Bars represent the group average mean absolute displacement ± SEM for each group. The average mean absolute displacemnt is computed for each subject seperately by averaging all head displacement in time. There were no significant differences. (TIF) [file pone.0106133.s003.tif]
