# Supplementary material for: Functional Reorganization of the Default Mode Network across Chronic Pain Conditions
Source: PLoS One. 2014 Sep 2;9(9):e106133. doi: 10.1371/journal.pone.0106133 (PMC4152156; doi:10.1371/journal.pone.0106133)
Supplement: Table S1 — Demographic and clinical data for CBP, CRPS and OA patients that participated in the fMRI study. BDI = Beck's depression inventory; M = male; F = female; VAS = visual analogue scale. The VAS was computed from the McGill short-form questionnaire (sf-MPQ). MQS is medication quantification questionnaire. (DOCX) [file pone.0106133.s004.docx]

| **Patient** | **Age**  **(years)** | **Gender** | BDI **(0- 40)** | **VAS**  **(0-10)** | **Duration**  **(years)** | **MQS** | **Medication** |
| --- | --- | --- | --- | --- | --- | --- | --- |
| CBP 1 | 51 | F | 15 | 8 | 1 | 34.8 | lyrica, mysoline, motrin, excedrin |
| CBP 2 | 62 | M | 1 | 8.7 | 38 | 5.7 | ibuprofen, motrin |
| CBP 3 | 49 | F | 12 | 8.4 | 14 | 44.9 | ultram, zanaflex, phenobarbital, Tylenol |
| CBP 4 | 62 | F | 13 | 8.4 | 2 | 17.5 | Ibuprofin, tylenol, darvocet |
| CBP 5 | 60 | F | 8 | 10 | 30 | 20.9 | xanax, morphin, tylenol |
| CBP 6 | 54 | F | 6 | 7.5 | 8 | 24 | ibuprofin, lyrica, darvocet, |
| CBP 7 | 50 | M | 4 | 6.9 | 30 | 12.5 | darvocet, tylenol |
| CBP 8 | 54 | M | 4 | 6.2 | 30 | 8.9 | Ibuprofen, tylenol |
| CBP 9 | 62 | M | 3 | 5.4 | 25 | 18.1 | morphine, mortin, ibuprofen |
| CBP 10 | 46 | M | 13 | 5.9 | 11 | 53.8 | xanax, vicodin, phenobarbital, tylenol, neurontin, ibuprofen |
| CBP 11 | 49 | M | 4 | 6.3 | 41 | 11.3 | mysoline, ibuprofen |
| CBP 12 | 52 | M | 6 | 5.7 | 5 | 6.9 | Ibuprofen, excedrin |
| CBP 13 | 54 | M | 4 | 6.2 | 30 | 27.3 | lyrica, mortin, lidoderm |
| CBP 14 | 58 | M | 9 | 8.4 | 10.7 | 0 |  |
| CBP 15 | 32 | M | 5 | 7.8 | 12 | 25.2 | darvocet, soma, ibuprofen |
| CBP 16 | 41 | M | 3 | 3 | 15 | 11.7 | trileptal, mortin |
| CBP 17 | 48 | M | 12 | 8.7 | 1 | 0 |  |
| CBP 18 | 44 | M | 0 | 2.8 | 18 | 0 |  |
| CRPS 1 | 61 | F | 6 | 8.2 | 0.25 | 16.8 | trileptal, celebrex, cymbalta, zanaflex, lyrica |
| CRPS 2 | 56 | M | 12 | 2.2 | 0.25 | 0 |  |
| CRPS 3 | 48 | M | 13 | 2.9 | 1.4 | 8.5 | lyrica, trileptal |
| CRPS 4 | 40 | M | -- | -- | -- | -- |  |
| CRPS 5 | 25 | F | 16 | 6.4 | 1.8 | 47.6 | neurontin, elavil, topamax, lidoderm, xanax, relpax, percocet, vicodin, morphine |
| CRPS 6 | 26 | F | 13 | 2 | 0.3 | 14.7 | lyrica, mysoline |
| CRPS 7 | 26 | F | 7 | 4.8 | 1 | 19.3 | zonegran, celebrex, relpax, protanix, timolol |
| CRPS 8 | 28 | F | 6 | 2.3 | 1.7 | 0 |  |
| CRPS 9 | 31 | F | 5 | 3.7 | 0.4 | 9.3 | trileptal, ultram, gabapentin |
| CRPS 10 | 32 | F | 5 | 1.5 | 6.1 | 62.2 | levothyroxin, lyrica, tramadol, darvocet, ibuprofen, xanaflex, cymbalta, rozerem, trileptal, methadone |
| CRPS 11 | 39 | F | 9 | 6.6 | 1.3 | 54.9 | lyrica, vicodin, neurontin,, methadone, ultram, ibuprofen, lidoderm, zanaflex |
| CRPS 12 | 40 | F | 10 | 8.2 | 13.5 | 27.5 | soma, lyrica, ambian cr, darvocet |
| CRPS 13 | 41 | F | 2 | 5.4 | -- | 0 |  |
| CRPS 14 | 43 | F | 14 | 5.6 | 5 | 35.6 | vicodin, lamictal, gabatril, trazodone, dibenzyline |
| CRPS 15 | 44 | F | 1 | 7.2 | 1.1 | 6.6 | trileptal, lyrica |
| CRPS 16 | 47 | F | 13 | 7.3 | 7.3 | 25.6 | methadone, cymbalta, zanaflex, lyrica |
| CRPS 17 | 48 | F | 4 | 6.9 | -- | 0 |  |
| CRPS 18 | 50 | F | 3 | 4.8 | 1 | 0 |  |
| CRPS 19 | 53 | F | 2 | 8.1 | 0.25 | 0 |  |
| OA 1 | 49 | M | 9 | 7.1 | 10 | 0 |  |
| OA 2 | 61 | F | 4 | 2 | 5 | 12.5 | mortin, ibuprofen |
| OA 3 | 58 | F | 4 | 7.5 | 15 | 45.3 | neurontin, xanax, ibuprofin, vicodin, morphine, tylenol |
| OA 4 | 63 | F | 1 | 5.8 | 1 | 23.7 | cymbalta, xanax, ibuprofen |
| OA 5 | 53 | M | 7 | 8.3 | 36 | 12.1 | mortin, lyrica |
| OA 6 | 66 | M | 1 | 2.1 | -- | 4.2 | ibuprofen |
| OA 7 | 55 | M | 2 | 7.3 | 20 | 0 |  |
| OA 8 | 51 | M | 22 | 9 | 14 | 0 |  |
| OA 9 | 56 | F | 13 | 5.6 | 6 | 3.7 | ibuprofen |
| OA 10 | 55 | M | 8 | 7.4 | 10 | 2.1 | ibuprofen |
| OA 11 | 42 | M | 9 | 5.7 | 5 | 0 |  |
| OA 12 | 53 | M | 0 | 7 | 11 | 22.4 | tylenol, Ibuprofin, morphine |
| OA 13 | 77 | F | 8 | 4.5 | 3 | 18.6 | morphine, mortin, ibuprofen |
| OA 14 | 77 | F | 8 | 5.8 | 7 | 0 |  |
