# Supplementary material for: Functional Reorganization of the Default Mode Network across Chronic Pain Conditions
Source: PLoS One. 2014 Sep 2;9(9):e106133. doi: 10.1371/journal.pone.0106133 (PMC4152156; doi:10.1371/journal.pone.0106133)
Supplement: Table S2 — Coordinates for peak foci for DMN ICA analysis. All coordinates listed in MNI space x, y, z values in mm.; MPFC = medial prefrontal cortex; PreCu = Precuneus; ACC = anterior cingulate cortex; LP = lateral parietal; IFG = Inferior frontal gyrus; INS = insula; SMG = supramarginal gyrus. (DOCX) [file pone.0106133.s005.docx]

| **Brain region** | **BA** | **Coordinates**  **x y z** | | | **F-zstat** |
| --- | --- | --- | --- | --- | --- |
| MPFC | 10 | -4 | 58 | 2 | 4.23 |
| PreCu | 23 | 2 | -56 | 26 | 3.65 |
| **ACC** | 24 | 2 | 36 | 22 | 5.54 |
| **Right LP** | 39 | 46 | -60 | 32 | 3.71 |
| **Left INS/IFG** | 38 | -38 | 10 | -12 | 3.05 |
| **Left SMG** | 48 | -56 | -36 | 26 | 3.32 |
