# Supplementary material for: Functional Reorganization of the Default Mode Network across Chronic Pain Conditions
Source: PLoS One. 2014 Sep 2;9(9):e106133. doi: 10.1371/journal.pone.0106133 (PMC4152156; doi:10.1371/journal.pone.0106133)
Supplement: Table S3 — Coordinates for peak foci for DMN ICA analysis without Gray matter correction. All coordinates listed in MNI space x, y, z values in mm; MPFC = medial prefrontal cortex; PreCu = Precuneus; ACC = anterior cingulate cortex; LP = lateral parietal; IFG = Inferior frontal gyrus; INS = insula; SMG = supramarginal gyrus; PCL = paracentral lobule; IPS = intra parietal sulcus. (DOCX) [file pone.0106133.s006.docx]

| **Brain region** | **BA** | **Coordinates**  **x y z** | | | **F-zstat** |
| --- | --- | --- | --- | --- | --- |
| MPFC | 10 | -6 | 50 | 6 | 3.28 |
| PreCu | 23 | 0 | -56 | 26 | 3.23 |
| **ACC** | 24 | 0 | 40 | 22 | 2.71 |
| **Left IPS** | 24 | -46 | -40 | 48 | 4.90 |
| **Right LP** | 39 | 46 | -60 | 30 | 3.92 |
| **PCL** | 4 | 4 | -28 | 12 | 4.35 |
| **Left IFG/INS** | 38 | -40 | 12 | -18 | 3.48 |
| **Left SMG** | 48 | -58 | -38 | 26 | 3.40 |
