# Supplementary material for: Functional Reorganization of the Default Mode Network across Chronic Pain Conditions
Source: PLoS One. 2014 Sep 2;9(9):e106133. doi: 10.1371/journal.pone.0106133 (PMC4152156; doi:10.1371/journal.pone.0106133)
Supplement: Table S4 — Relationship between DMN properties and clinical parameters in patients. Correlation between pain intensity or duration with DMN size, high frequency (HF) power and Δphase for all groups of patients computed seperately (data from Figure 4) [* p<0.05, ** p<0.01]. (DOCX) [file pone.0106133.s007.docx]

|  | **Pain duration (years)** | | | Pain intensity (VAS) | | |
| --- | --- | --- | --- | --- | --- | --- |
|  | ***CBP*** | ***CRPS*** | *OA* | ***CBP*** | ***CRPS*** | ***OA*** |
| DMN size | 0.15 | 0.07 | -0.10 | -0.47 | 0.20 | -0.01 |
| **DMN HF power** | 0.65** | 0.11 | 0.77** | 0.11 | -0.22 | -0.19 |
| **DMN** Δ**phase** | 0.68* | 0.19 | 0.64 | 0.43 | 0.28 | 0.13 |
