# Supplementary material for: Functional Reorganization of the Default Mode Network across Chronic Pain Conditions
Source: PLoS One. 2014 Sep 2;9(9):e106133. doi: 10.1371/journal.pone.0106133 (PMC4152156; doi:10.1371/journal.pone.0106133)
Supplement: Table S5 — Relationship between mean absolute displacement and all functional paramters assessed in the study. There was no signifecent correlation with head motion and functional connectivity parameters across all groups. (DOCX) [file pone.0106133.s008.docx]

|  |  | **Healthy** | | **CBP** | | **CRPS** | | **OA** | |
| --- | --- | --- | --- | --- | --- | --- | --- | --- | --- |
|  |  | **R** | **p-value** | **R** | **p-value** | **R** | **p-value** | **R** | **p-value** |
| **Size**  **(Figure 1)** | **DMN** | -0.02 | 0.91 | 0.22 | 0.38 | 0.06 | 0.80 | 0.01 | 0.96 |
|  | **Salience** | -0.17 | 0.32 | 0.11 | 0.65 | -0.21 | 0.39 | 0.03 | 0.91 |
|  | **Sensorimotor** | 0.02 | 0.91 | 0.25 | 0.32 | -0.18 | 0.45 | -0.39 | 0.17 |
|  | **Frontoparietal** | 0.02 | 0.92 | 0.31 | 0.21 | 0.25 | 0.29 | 0.08 | 0.78 |
|  | **Visual** | -0.21 | 0.22 | -0.10 | 0.68 | -0.14 | 0.57 | -0.38 | 0.18 |
| **ICA analysis**  **(Figure 2)** | **ACC** | 0.30 | 0.07 | -0.14 | 0.57 | 0.32 | 0.19 | 0.21 | 0.47 |
|  | **right LP** | -0.17 | 0.32 | 0.30 | 0.23 | 0.06 | 0.79 | -0.08 | 0.80 |
|  | **left INS/IFG** | 0.04 | 0.82 | 0.32 | 0.20 | 0.03 | 0.91 | 0.29 | 0.32 |
|  | **MPFC** | 0.20 | 0.25 | -0.39 | 0.11 | -0.03 | 0.90 | -0.14 | 0.64 |
|  | **PreCu** | -0.07 | 0.69 | -0.31 | 0.21 | -0.06 | 0.82 | -0.14 | 0.63 |
|  | **Left SMG** | 0.22 | 0.19 | 0.09 | 0.73 | 0.26 | 0.28 | 0.44 | 0.12 |
| **Frequency & phase analysis**  **(Figure 3)** | **DMN HF power** | 0.12 | 0.50 | 0.13 | 0.61 | -0.04 | 0.89 | 0.14 | 0.63 |
|  | **MPFC HF power** | 0.09 | 0.60 | 0.28 | 0.26 | 0.29 | 0.24 | -0.01 | 0.99 |
|  | **PreCu HF power** | -0.09 | 0.61 | 0.44 | 0.06 | 0.17 | 0.50 | -0.22 | 0.45 |
|  | **right LP HF power** | 0.00 | 0.98 | 0.22 | 0.37 | 0.03 | 0.90 | -0.32 | 0.27 |
|  | **DMN** Δ**phase** | 0.17 | 0.33 | -0.28 | 0.26 | -0.19 | 0.43 | -0.05 | 0.87 |
| **Correlation analysis**  **(Figure 5)** | **DMN - MPFC** | -0.13 | 0.45 | -0.41 | 0.09 | 0.09 | 0.72 | -0.22 | 0.44 |
|  | **DMN - PreCu** | -0.05 | 0.78 | -0.29 | 0.25 | -0.32 | 0.18 | -0.26 | 0.38 |
|  | **DMN - right LP** | -0.14 | 0.43 | -0.18 | 0.48 | 0.02 | 0.93 | 0.30 | 0.29 |
|  | **DMN - left LP** | 0.13 | 0.46 | 0.18 | 0.48 | -0.18 | 0.47 | -0.18 | 0.55 |
|  | **MPFC - PreCu** | -0.33 | 0.05 | -0.30 | 0.22 | -0.27 | 0.26 | -0.37 | 0.20 |
|  | **MPFC - INS** | 0.05 | 0.76 | 0.22 | 0.37 | 0.02 | 0.94 | -0.06 | 0.85 |
