# Supplementary material for: Functional Reorganization of the Default Mode Network across Chronic Pain Conditions
Source: PLoS One. 2014 Sep 2;9(9):e106133. doi: 10.1371/journal.pone.0106133 (PMC4152156; doi:10.1371/journal.pone.0106133)
Supplement: Table S6 — Relationship between Drug usage and all functional paramters assessed in the study. Except for DMN-left LP correlation in CBP and sensorimotor size in CRPS, there was no signifecent association between drug usage and all functional parameters assessed in the study. (significent relationships are shown in red). MQS is a validated pain medication use questionnaire, which generates equivalences between various analgesic drugs. (DOCX) [file pone.0106133.s009.docx]

|  |  | **CBP** | | **CRPS** | | **OA** | |
| --- | --- | --- | --- | --- | --- | --- | --- |
|  |  | **R** | **p-value** | **R** | **p-value** | **R** | **p-value** |
| **Size analysis**  **(Figure 1)** | **DMN** | -0.37 | 0.13 | 0.19 | 0.45 | -0.29 | 0.31 |
|  | **Salience** | 0.35 | 0.15 | 0.43 | 0.07 | 0.52 | 0.05 |
|  | **Sensorimotor** | -0.13 | 0.60 | 0.56 | <0.05 | 0.47 | 0.09 |
|  | **Frontoparietal** | -0.05 | 0.85 | 0.06 | 0.80 | 0.24 | 0.40 |
|  | **Visual** | -0.34 | 0.16 | -0.25 | 0.31 | -0.10 | 0.73 |
| **ICA analysis**  **(Figure 2)** | **ACC** | 0.40 | 0.10 | -0.34 | 0.17 | -0.04 | 0.89 |
|  | **right LP** | 0.07 | 0.79 | 0.13 | 0.59 | 0.20 | 0.49 |
|  | **left INS/IFG** | -0.47 | 0.05 | 0.24 | 0.34 | 0.14 | 0.63 |
|  | **MPFC** | 0.04 | 0.86 | -0.11 | 0.66 | -0.44 | 0.11 |
|  | **PreCu** | 0.01 | 0.98 | 0.20 | 0.43 | 0.30 | 0.29 |
|  | **Left SMG** | -0.21 | 0.41 | 0.21 | 0.40 | -0.32 | 0.26 |
| **Frequency & phase analysis**  **(Figure 3)** | **DMN HF power** | -0.17 | 0.51 | -0.02 | 0.94 | -0.15 | 0.60 |
|  | **MPFC HF power** | -0.03 | 0.92 | 0.07 | 0.78 | -0.44 | 0.11 |
|  | **PreCu HF power** | -0.27 | 0.27 | 0.05 | 0.84 | -0.40 | 0.16 |
|  | **right LP HF power** | -0.25 | 0.32 | 0.10 | 0.71 | -0.14 | 0.62 |
|  | **DMN** Δ**phase** | 0.17 | 0.50 | -0.11 | 0.66 | 0.35 | 0.22 |
| **Correlation analysis**  **(Figure 5)** | **DMN - MPFC** | -0.15 | 0.54 | -0.34 | 0.17 | 0.06 | 0.84 |
|  | **DMN - PreCu** | 0.11 | 0.66 | 0.00 | 0.98 | 0.35 | 0.22 |
|  | **DMN - right LP** | 0.09 | 0.73 | 0.04 | 0.87 | 0.18 | 0.53 |
|  | **DMN - left LP** | 0.57 | <0.05 | 0.05 | 0.84 | 0.20 | 0.50 |
|  | **MPFC - PreCu** | 0.14 | 0.58 | -0.32 | 0.20 | 0.28 | 0.32 |
|  | **MPFC - INS** | -0.19 | 0.44 | 0.12 | 0.64 | -0.43 | 0.12 |
