# Supplementary material for: Functional Reorganization of the Default Mode Network across Chronic Pain Conditions
Source: PLoS One. 2014 Sep 2;9(9):e106133. doi: 10.1371/journal.pone.0106133 (PMC4152156; doi:10.1371/journal.pone.0106133)
Supplement: Table S7 — Relationship between depression and all functional paramters assessed in the study. Except for left SMG connectvity and sensorimotor size in OA, there was no signifecent association between depression, measured used the Beck's deprresion index, and all functional parameters assessed in the study. (significent relationships are shown in red). (DOCX) [file pone.0106133.s010.docx]

|  |  | **Healthy** | | **CBP** | | **CRPS** | | **OA** | |
| --- | --- | --- | --- | --- | --- | --- | --- | --- | --- |
|  |  | **R** | **p-value** | **R** | **p-value** | **R** | **p-value** | **R** | **p-value** |
| **Size**  **(Figure 1)** | **DMN** | 0.20 | 0.25 | -0.02 | 0.93 | -0.03 | 0.90 | 0.36 | 0.21 |
|  | **Salience** | 0.22 | 0.20 | -0.24 | 0.35 | 0.28 | 0.27 | -0.13 | 0.66 |
|  | **Sensorimotor** | -0.20 | 0.25 | -0.44 | 0.06 | 0.05 | 0.83 | -0.63 | <0.05 |
|  | **Frontoparietal** | 0.03 | 0.85 | 0.45 | 0.06 | 0.17 | 0.51 | 0.10 | 0.74 |
|  | **Visual** | -0.11 | 0.52 | -0.17 | 0.49 | -0.34 | 0.17 | -0.52 | 0.06 |
| **ICA analysis**  **(Figure 2)** | **ACC** | 0.05 | 0.76 | 0.13 | 0.62 | -0.15 | 0.55 | 0.14 | 0.63 |
|  | **right LP** | 0.03 | 0.86 | 0.03 | 0.90 | 0.29 | 0.24 | 0.11 | 0.72 |
|  | **Left INS/IFG** | -0.18 | 0.28 | -0.34 | 0.17 | -0.03 | 0.90 | 0.08 | 0.79 |
|  | **MPFC** | 0.06 | 0.74 | 0.01 | 0.98 | 0.00 | 0.99 | -0.13 | 0.65 |
|  | **PreCu** | 0.05 | 0.77 | -0.05 | 0.83 | 0.00 | 0.99 | -0.04 | 0.90 |
|  | **Left SMG** | 0.06 | 0.72 | 0.11 | 0.66 | 0.17 | 0.50 | 0.66 | <0.01 |
| **Frequency & phase analysis**  **(Figure 3)** | **DMN HF power** | 0.04 | 0.80 | 0.08 | 0.76 | -0.28 | 0.26 | -0.24 | 0.40 |
|  | **MPFC HF power** | 0.09 | 0.59 | -0.15 | 0.56 | -0.23 | 0.35 | -0.10 | 0.73 |
|  | **PreCu HF power** | -0.03 | 0.87 | -0.12 | 0.63 | -0.17 | 0.50 | 0.03 | 0.92 |
|  | **right LP HF power** | 0.06 | 0.72 | 0.05 | 0.83 | -0.40 | 0.10 | -0.13 | 0.65 |
|  | **DMN** Δ**phase** | 0.10 | 0.56 | 0.20 | 0.43 | -0.17 | 0.51 | -0.14 | 0.64 |
| **Correlation analysis**  **(Figure 5)** | **DMN - MPFC** | -0.20 | 0.23 | -0.38 | 0.12 | -0.32 | 0.19 | -0.12 | 0.69 |
|  | **DMN - PreCu** | -0.05 | 0.79 | 0.13 | 0.60 | 0.08 | 0.76 | -0.22 | 0.45 |
|  | **DMN - right LP** | -0.09 | 0.59 | 0.03 | 0.90 | 0.24 | 0.34 | 0.32 | 0.26 |
|  | **DMN - left LP** | 0.07 | 0.67 | 0.00 | 1.00 | 0.14 | 0.58 | -0.27 | 0.35 |
|  | **MPFC - PreCu** | -0.23 | 0.17 | -0.09 | 0.71 | -0.28 | 0.26 | -0.27 | 0.35 |
|  | **MPFC - INS** | 0.11 | 0.51 | -0.02 | 0.93 | 0.27 | 0.27 | -0.03 | 0.93 |
